# A549 Alveolar Carcinoma Spheroids as a Cytotoxicity Platform for Carboxyl‐ and Amine‐Polyethylene Glycol Gold Nanoparticles

**DOI:** 10.1002/prp2.70051

**Published:** 2024-12-26

**Authors:** Melissa Petzer, Seth‐Frerich Fobian, Mary Gulumian, Vanessa Steenkamp, Werner Cordier

**Affiliations:** ^1^ Department of Pharmacology, Faculty of Health Sciences University of Pretoria Pretoria South Africa; ^2^ Molecular Medicine and Haematology, School of Pathology University of Witwatersrand Johannesburg South Africa; ^3^ Water Research Group, Unit for Environmental Sciences and Management North‐West University Potchefstroom South Africa

**Keywords:** 3D culture, alveolar carcinoma, cytotoxicity, gold nanoparticles, spheroid, spheroid model

## Abstract

Gold nanoparticles (AuNPs) present with unique physicochemical features and potential for functionalization as anticancer agents. Three‐dimensional spheroid models can be used to afford greater tissue representation due to their heterogeneous phenotype and complex molecular architecture. This study developed an A549 alveolar carcinoma spheroid model for cytotoxicity assessment and mechanistic evaluation of functionalized AuNPs. A549 spheroids were generated using an agarose micro‐mold and were characterized (morphology, acid phosphatase activity, protein content) over 21 culturing days. The 72‐h cytotoxicity of carboxyl‐polyethylene glycol‐ (PCOOH‐) and amine‐polyethylene glycol‐ (PNH_2_‐) functionalized AuNPs against Day 7 spheroids was assessed by determining spheroid morphology, acid phosphatase activity, protein content, caspase‐3/7 activity, and cell cycle kinetics. Spheroids remained stable over the experimental period. Although the A549 spheroids' volume increased while remaining viable over the culturing period, structural integrity decreased from Day 14 onwards. The PCOOH‐AuNPs lacked cytotoxicity at a maximum concentration of 1.2 × 10^12^ nanoparticles/mL with no prominent alteration to the cellular processes investigated, while the PNH_2_‐AuNPs (at a maximum of 4.5 × 10^12^ nanoparticles/mL) displayed dose‐ and time‐dependent cytotoxicity with associated loss of spheroid compactness, debris formation, DNA fragmentation, and a 75% reduction in acid phosphatase activity. Differentiation between cytotoxic and non‐cytotoxic AuNPs was achieved, with preliminary elucidation of cytotoxicity endpoints. The PNH_2_‐AuNPs promote cytotoxicity by modulating cellular kinetics while destabilizing the spheroid ultrastructure. The model serves as a proficient platform for more in‐depth elucidation of NP cytotoxicity at the preclinical investigation phase.

## Graphical abstract

Cytotoxicity of amine‐polyethylene glycol‐functionalised gold nanoparticles in A549 spheroids in comparison to non‐cytotoxic carboxyl‐polyethylene glycol‐functionalised gold nanoparticles.

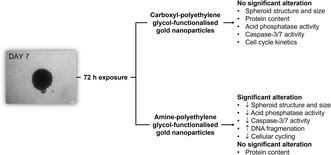

## 1 Introduction

Lung cancer is considered a large contributor to cancer‐related mortality worldwide [1], with an estimated 238 340 new cases diagnosed in the United States in 2023 [2]. According to Statistics South Africa (2008 to 2019 report), lung cancer was the leading cause of cancer‐related mortality in South Africa, being the third‐most diagnosed cancer in males and the seventh‐most diagnosed cancer in females [3]. Although there are several treatments available, the diagnosis and chemotherapeutic treatment of lung cancer remain challenging [4].

Chemoresistance is a major obstruction in cancer treatment [5, 6], with acquired resistance being evident in several cancer types [7]. Tumors also possess innate resistance to chemotherapy [7] due to physical barriers preventing drug penetration through the cellular layers [8]. Cancer cells further alter cell cycling and apoptotic processes to prevent chemotherapeutic‐induced cytotoxicity [9], which contributes to the preferential targeting of actively proliferating cells [10]. The extracellular matrix also alters cellular signalling [9, 11] to protect solid tumors [5] and modulate cellular proliferation and differentiation in response to chemotherapy [8].

One promising way to address and overcome these obstacles is to improve the delivery of chemotherapy to the tumor by employing nanomedicine [8]. Nanomedicine, which uses nanoparticles (NPs) for a range of theragnostic ends, is an attractive field of study for drug delivery and directed therapy [12]. In oncopharmacotherapy, for example, the non‐specific nature of chemotherapeutic drugs, leading to the systemic circulation thereof, often leads to severe adverse effects [13]. However, by employing NPs with specific functional groups, a more targeted approach is achieved [14, 15]. This allows for greater delivery of drugs to targeted organs; reduction of non‐specific effects; improved efficacy; and a decrease in side effects [12]. Altering the physicochemical properties allows for more amenable solubility, in vivo stability, and biodistribution of the drug [16]. However, concerns have been raised as to the toxicity of NPs, therefore necessitating cytotoxicity screening [17, 18, 19]. Without functionalization, NPs may present with non‐specificity and the inability to reach the desired target, resulting in excessive and non‐specific cytotoxicity [17]. For example, oxidative stress has been observed for some NPs in keratinocytes [20], macrophages, and monocytes [21]. Additionally, inflammation may occur in exposed cells containing chemically inactive NPs [22].

Gold nanoparticles (AuNPs) are frequently studied in medical applications [23, 24] due to their promise as therapeutic delivery vehicles. The AuNPs have several appealing qualities, including unique photo‐optical properties for biomedical optical imaging [24, 25] and sensitization properties in radiation therapy [26]. Although some AuNPs lack cytotoxicity [16, 27, 28], biocompatibility still needs to be considered during drug development, and these compounds cannot be assumed safe until proven so [22]. Cells might be exposed to the AuNPs for extended periods of time due to particle internalization [27, 29], and AuNPs cause cellular damage via the induction of oxidative stress and subsequent up‐regulation of inflammatory genes, highlighting the need for cytotoxicity screening [30].

The A549 alveolar carcinoma cell line was selected for experimental assays as continuation of previous research by the group [31], seeing that 3D culturing was known to be successful [32, 33] and that cellular uptake does occur [34]. The study aimed to modify and characterize an A549 alveolar carcinoma spheroid model for AuNP‐induced cytotoxicity assessment using 14 nm carboxyl‐polyethylene glycol (PCOOH)‐liganded AuNPs (PCOOH‐AuNPs) and 20 nm amine‐polyethylene glycol (PNH_2_)‐liganded AuNPs (PNH_2_‐AuNPs).

## 2 Materials and Methods

### 2.1 Reagents

A549 alveolar carcinoma cells (CCL‐185) were procured from the American Type Culture Collection (ATCC; Manassas, USA). Gibco Dulbecco's modified Eagle medium (DMEM) and TrypLe were purchased from ThermoFisher (Johannesburg, South Africa). The PCOOH‐AuNPs and PNH_2_‐AuNPs were obtained from Mintek (Randburg, South Africa). Accutase solution, propidium iodide (PI), all salts required for preparation of bicinchoninic acid (BCA) assay reagent A and B, radioimmunoprecipitation assay (RIPA) buffer, bovine serum albumin (BSA) powder, fetal calf serum (FCS), penicillin/streptomycin solution, agarose powder, 9 × 9 array negative polydimethylsiloxane molds (MicroTissues 3D Petri Dish), sodium citrate dihydrate, citric acid, saponin, Ac‐DEVD‐AMC powder, 4‐(2‐hydroxyethyl)‐1‐piperazineethanesulfonic acid (HEPES) powder, ethylenediaminetetraacetic acid (EDTA), 3‐[(3‐cholamidopropyl)‐dimethylammonio]‐1‐propanesulfate (CHAPS), β‐mercaptoethanol, and cisplatin were obtained from Merck (Johannesburg, South Africa). The FTA hemagglutination buffer was purchased from BD Biosciences (Sandton, South Africa). The phenylmethylsulphonyl fluoride (PMSF) powder was procured from Sigma‐Aldrich (St. Louis, USA).

### 2.2 Nanoparticles

#### 2.2.1 Selection of Nanoparticles

Two AuNPs were selected: 14 nm PCOOH‐AuNPs and 20 nm PNH_2_‐AuNPs. The PCOOH‐AuNPs have previously been shown to lack cytotoxicity and uptake across multiple cell layers of the A549 spheroid model [34], and thus were employed as a non‐cytotoxic control.

#### 2.2.2 Synthesis and Characterization

The 14 nm PCOOH‐AuNPs were prepared by Mintek (South Africa) by ligand exchange of citrate‐stabilized AuNPs with 3 kDA thiolated‐PEG‐COOH [31] according to published methods [35, 36]. The same process was followed for the synthesis of 20 nm amine‐polyethylene glycol (NH_2_)‐liganded AuNPs (PNH_2_‐AuNPs), where in this case 3 kDa thiolated‐PEG (HS‐PEG‐NH_2_) was used for ligand exchange. Interference of AuNPs with the wavelengths and substrates was determined using all experimental settings referred to below in an acellular environment. Little to no interference was noted (Data S1).

### 2.3 Cell Culture and Maintenance

#### 2.3.1 Spheroid Generation

A549 (ATCC CCL‐185) spheroids were generated using a modification of the liquid overlay method employed by Fobian et al. [34] using a 9 × 9 array negative polydimethylsiloxane mold. Molds were autoclaved prior to each microwell generation to ensure sterility. To create the microwell system, a sterile liquid agarose solution (1.6%, 500 μL) was pipetted into the mold to create an agarose platform with 81 microwells. Each mold was inserted into a 12‐well plate, and 160 μL of cell suspension (3.8 × 10^6^ cells/mL) was pipetted into the mold. Plates were incubated at 37°C and 5% CO_2_ for 24 h while avoiding unnecessary turbulence. After 24 h, DMEM (1 mL) was pipetted alongside the mold to ensure nutrient transfer. Medium was replenished every 4 to 7 days after the spheroids had formed. The spheroids were grown for up to 21 days, with formation, growth, and viability assessed at regular intervals (Days 4, 7, 11, 14, 18, and 21).

### 2.4 Characterization of Spheroids

#### 2.4.1 Morphological and Planimetric Characterization

A Carl Zeiss Axiovert 200 M inverted microscope (Carl Zeiss Inc., Oberkochen, Germany) at a 5× magnification was used to visualize the A549 spheroids on Days 4, 7, 11, 14, 18, and 21. Micrographs were taken at selected intervals to determine the volume, circularity, and diameter of the spheroids using ImageJ by using the equations:

$$\text{Spheroid volume}\;\left(V\right)=0.5\times \left(\text{length}\right)\times {\left(\text{width}\right)}^{2}$$

$$\%\text{change in spheroid volume}=\frac{V\;\mathrm{on}\;\mathrm{day}\;\left[\#\right]-V\;\mathrm{on}\;\mathrm{Day}\;4}{V\;\mathrm{on}\;\mathrm{Day}\;4}\times 100$$

$$\text{Circularity}=4\pi \;\left(\frac{\text{Area}}{{\text{Perimeter}}^{2}}\right)$$

#### 2.4.2 Viability Characterization

Acid phosphatases (APH) are lysosomal enzymes that hydrolyze organic phosphates in acidic environments. A proportional decrease is associated with cytotoxicity. Washed, pooled spheroids (*n* = 3) were re‐suspended in 100 μL PBS and 100 μL assay buffer (containing para‐nitrophenylphosphate [4 mg/mL] and Triton X‐100 [0.2% v/v] in citrate buffer [0.1 M]) at 37°C for 90 min. After incubation, 10 μL sodium hydroxide (1 M) was added to each well to stop the reaction. The absorbance was read at 405 nm (reference: 630 nm) with an ELX800 UV microplate reader (BioTek Instruments Inc., Highland Park, USA) within 10 min of adding sodium hydroxide. All the absorbance values were blank‐subtracted, and the APH activity was calculated as a percentage using the following formula:

$$\mathrm{APH}\;\text{activity}\;\left(\%\text{relative to}\;\mathrm{Day}\;4\right)=\frac{\mathrm{OD}\;\text{sample}}{\text{Average}\;\mathrm{OD}\;\text{of}\;\mathrm{Day}\;4}\times 100$$

#### 2.4.3 Protein Content Determination

The BCA assay was used to quantify the amount of protein present in the spheroids, where the spectrophotometric intensity of the product correlates to protein concentration. Eight spheroids were pooled and washed twice with PBS (100 μL; 200 *g* for 5 min). The spheroids were lysed using 100 μL RIPA buffer (50 mM Tris‐hydrochloride [pH 7.4], 150 mM sodium chloride, 1% Triton X‐100, 1% sodium deoxycholate, 0.1% w/v sodium dodecyl sulphate, 1 mM EDTA, and 0.02% w/v Roche complete protease inhibitor cocktail). The spheroids were vortex‐mixed, sonicated on ice for 5 min, and centrifuged at 16 000 × *g* for 10 min. The lysate (as supernatant) was collected and stored at −80°C until use.

A clear 96‐well flat‐bottom plate was used to conduct the assay. An aliquot (5 μL) of the standard (BSA; 0.1 to 2 mg/mL in PBS) or spheroid lysate was added to the wells with 195 μL BCA working solution. The plate was shaken for 10 min at room temperature and incubated at 60°C for 30 min. The plate was cooled to room temperature, and the absorbance was measured with an ELX800UV microplate reader at 570 nm. Lysate protein content (mg/mL) was interpolated from the BSA standard curve, and dilutions were considered to determine individual spheroid content (μg/spheroid). The percentage change in the protein content was calculated in relation to the protein content on previous days. For example, the change in the percentage protein content between Day 4 and Day 7 was calculated using the formula:

$$\%\text{change in protein content}=\frac{\text{Protein}\;\left(\mathrm{Day}\;7\right)-\text{Protein}\;\left(\mathrm{Day}\;4\right)}{\text{Protein}\;\left(\mathrm{Day}\;4\right)}\times 100$$

#### 2.4.4 Gold Nanoparticle Exposure

The spheroids were deemed appropriate to use at Day 7 as they were compact and maintained their morphological features, which corresponded with previous uptake studies done by the research team [34]. Mature spheroids were carefully removed from the mold and placed into a standard liquid‐overlay 96‐well plate. The spheroids were exposed to 100 μL of either PCOOH‐AuNPs (6 × 10^11^ NP/mL, 1.2 × 10^12^ NP/mL) or PNH_2_‐AuNPs (1.1 × 10^12^ NP/mL PNH_2_‐AuNPs, 2.3 × 10^12^ NP/mL, 4.5 × 10^12^ NP/mL) for 24, 48, and 72 h at 37°C and 5% CO_2_.

#### 2.4.5 Effects of Gold Nanoparticles on Spheroids

The effects of AuNPs on morphology, planimetry, viability, and protein content were determined after each exposure time using the aforementioned methods. The negative and vehicle controls included FCS‐free DMEM and 1% dimethyl sulfoxide, respectively. Positive controls included saponin (1%, cytotoxicity), cisplatin (100 μM, cytotoxicity and caspase‐3/7), FCS depletion (24 h G_0_/G_1_‐phase block), methotrexate (40 μM, 24 h S‐phase block), and curcumin (24 h, 80 μM G_2_/M‐phase block).

#### 2.4.6 Gold Nanoparticle Effect on Caspase‐3/7 Activity as a Surrogate for Apoptosis

As a surrogate for potential induction of apoptosis, caspase‐3/7 activity was measured using the acetyl‐Asp‐Glu‐Val‐Asp‐7‐amido‐4‐methylcoumarin (Ac‐DEVD‐AMC) conversion assay. Ac‐DEVD‐AMC is a synthetic tetrapeptide substrate that is cleaved by activated caspases‐3/7 to free the bound fluorogenic 7‐amido‐4‐coumarin (AMC). As caspase‐3/7 is only activated via pro‐apoptotic pathways, fluorescence indicates an induction of programmed cell death. Four pooled spheroids were placed in a 1.5 mL tube and washed with PBS (1 mL) twice via centrifugation (200 *g* for 5 min). Cold lysis buffer (100 μL) was added to the spheroids and incubated on ice with gentle aspiration every 10 min until lysis was observed (approximately 30 min). The cell lysate (25 μL) was pipetted into the well of a black‐walled, clear‐bottom 96‐well plate, and Ac‐DEVD‐AMC‐containing substrate buffer was added (100 μL). The plate was incubated at 37°C for 4 h, and the fluorescence intensity (FI) was measured at an excitation and emission wavelength of 340 and 450 nm, respectively. The caspase‐3/7 activity was calculated as follows:

$$\text{Caspase}-3/7\;\text{activity}\;\left(\text{fold}-\text{change}\right)=\frac{\mathrm{FI}\;\text{sample}}{\mathrm{FI}\;\text{control average}}$$

#### 2.4.7 Cell Cycle Distribution Analysis

Flow cytometry‐mediated cell cycle analysis enables the quantification of cellular distribution in the different phases of the cell cycle based on differential propidium iodide staining. To yield single‐cell samples, the spheroids were dissociated with minor modifications to volumes and incubation periods. Thirty spheroids were pooled in a 1.5 mL tube and suspended in PBS (1 mL). Pooled spheroids were washed thrice via centrifugation (200 × *g* for 5 min). The spheroids were dissociated using 900 μL Accutase and gently mixed by aspiration. The tube was placed in a 37°C heated plate shaker for 10 min, after which the mixture was gently aspirated ten times to facilitate dissociation. The dissociated cellular suspension was centrifuged at 200 *g* for 5 min, after which the supernatant was decanted, and the pellet re‐suspended in FCS‐supplemented PBS (1%, 600 μL). While vortex‐mixing, ice‐cold absolute ethanol (1.4 mL) was added to the tube in a dropwise fashion to fix the cells. The solution was incubated overnight in a refrigerator. Fixed cells were washed and re‐suspended in 500 μL staining solution (80 μg/mL PI, 0.1% Triton X‐100, and 100 μg/mL DNA‐free RNase in PBS) for 40 min at 37°C. The samples were analyzed using a Beckman CytoFLEX flow cytometer (Beckman Coulter, South Africa). Deconvolution software (Kaluza) was used to measure DNA distribution, and the CytoExploreR (R‐script) was used to analyze data. Cells were classified in different phases of the cell cycle (G_0_/G_1−_, S‐, and G_2_/M‐phase), with the sub‐G_1_‐phase indicative of DNA fragmentation due to endonuclease activity associated with apoptosis.

### 2.5 Statistical Analysis

Each experiment was carried out using a minimum of three biological and technical replicates, allowing for at least nine data points to be generated per sample. Data on the diameter and volume of the spheroids obtained from microscopy results was processed using ImageJ. Results were reported as the mean ± the standard error of the mean (SEM). Data from the results of all assays was captured using Microsoft Excel and analyzed statistically using GraphPad Prism 5. Statistical analyses for all assays, apart from the cell cycle distribution, were done by performing a Kruskall‐Wallis test with post hoc Dunn's test. Cell cycle results obtained from flow cytometry were analyzed using two‐way analysis of variance facilitated by R with the use of CytoExploreR, an R script developed by Hammill [37]. Significance was considered as *p* < 0.05.

## 3 Results

### 3.1 Establishing a 3D Model for A549 Spheroids

**FIGURE 1:**
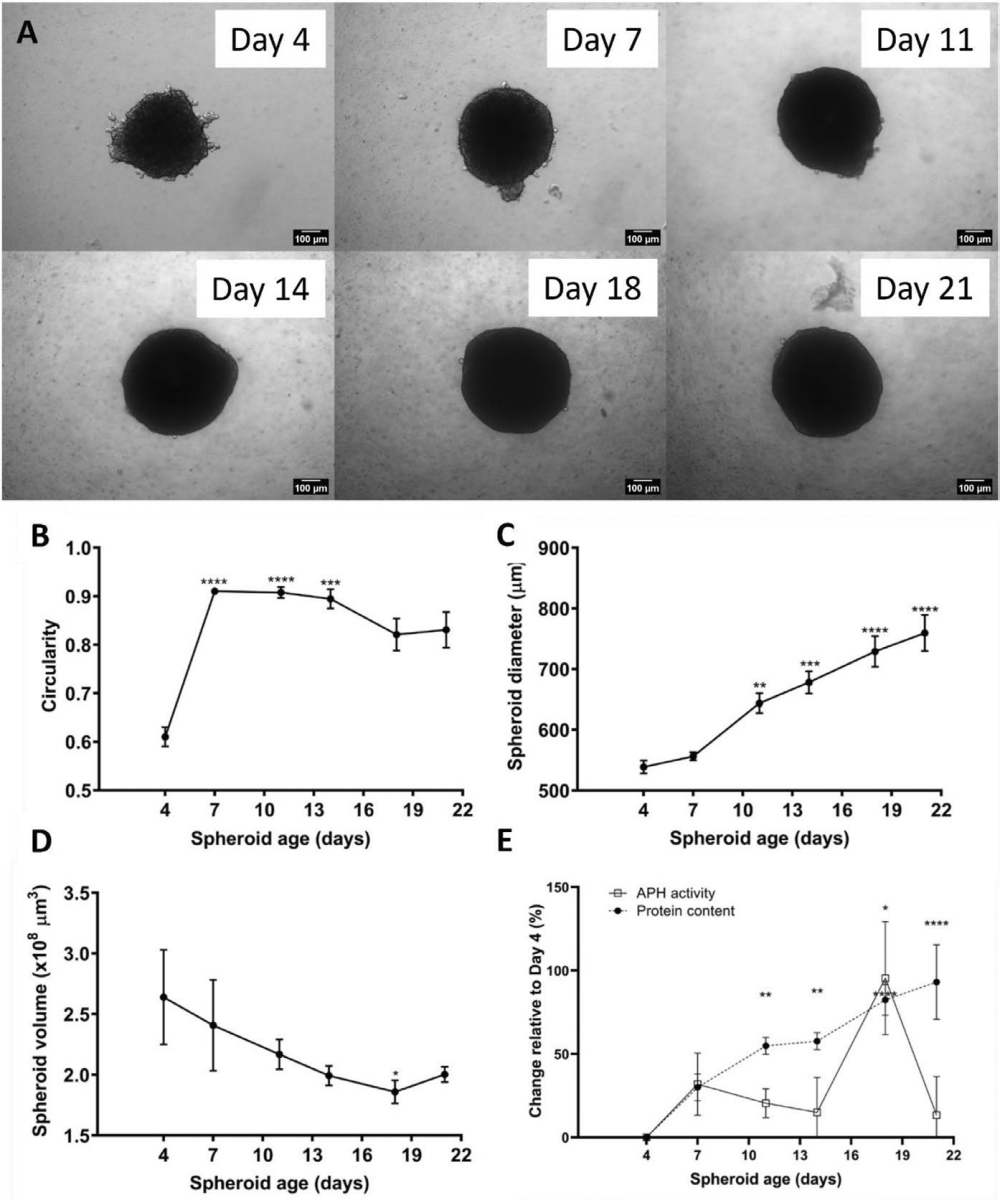
The growth of A549 spheroids over 21 days. Phase‐contrast microscopy images where Day 7 is indicative of the exposure time for nanoparticles in later experiments (5× objective; scale bar = 100 μm; A). Changes in spheroid circularity (B), diameter (C), volume (D), and acid phosphatase and protein content (E). Significance indicated as *p ≤ 0.05; **p ≤ 0.01; ***p ≤ 0.001; ****p ≤ 0.0001 as compared to Day 4.

The A549 spheroids were successfully formed by Day 4 when using the micro‐mold liquid overlay method and were maintained up to Day 21, as supported by microscopy images (Figure 1A). There was a significant (*p* < 0.0001) increase in circularity from Day 4 (0.6) to Day 7 (0.9) (Figure 1B), after which the spheroids remained stable till Day 14. From Days 14 to 18, there was a steady decrease in circularity to 0.8, which stabilized until Day 21. Microscopy confirmed the clearly defined spheroid edges and high opacity, indicative of compaction (Figure 1B). The spheroid volume initially decreased from Day 4 (2.64 × 10^8^ μm^3^) to Day 18 (1.86 × 10^8^ μm^3^; *p* < 0.05), before marginally increasing to 2.00 × 10^8^ μm^3^ on Day 21 (Figure 1C). Spheroid diameter was relatively unchanged from Day 4 (538.71 μm) to Day 7, after which a significant increase (*p* < 0.001) was observed until Day 22 (759.61 μm) (Figure 1D). Day 7 was regarded as the optimal day for AuNP exposure since the spheroids were fully formed and displayed the least variability in diameter, volume, and circularity for the three‐day maximum exposure period. Additionally, spheroids maintained structural integrity and viability for long enough to permit exposure to AuNPs for 72 h. The acid phosphatase (APH) activity initially increased by 31.9% from Day 4 (0.11) to Day 7 (0.14), after which it plateaued until Day 14 (Figure 1E). A sharp increase of 95.4% in activity was observed at Day 18 (0.21), compared to Day 4, which decreased to an activity similar to what was observed for Day 11 (0.13). Protein content initially increased by 30.0% from Day 4 (2.5 μg) to Day 7 (3.2 μg) and continued to increase until Day 21 (0.59 μg) (Figure 1E).

### 3.2 Effects of Gold Nanoparticle Exposure on A549 Spheroids

#### 3.2.1 PCOOH‐Liganded Gold Nanoparticles

**FIGURE 2:**
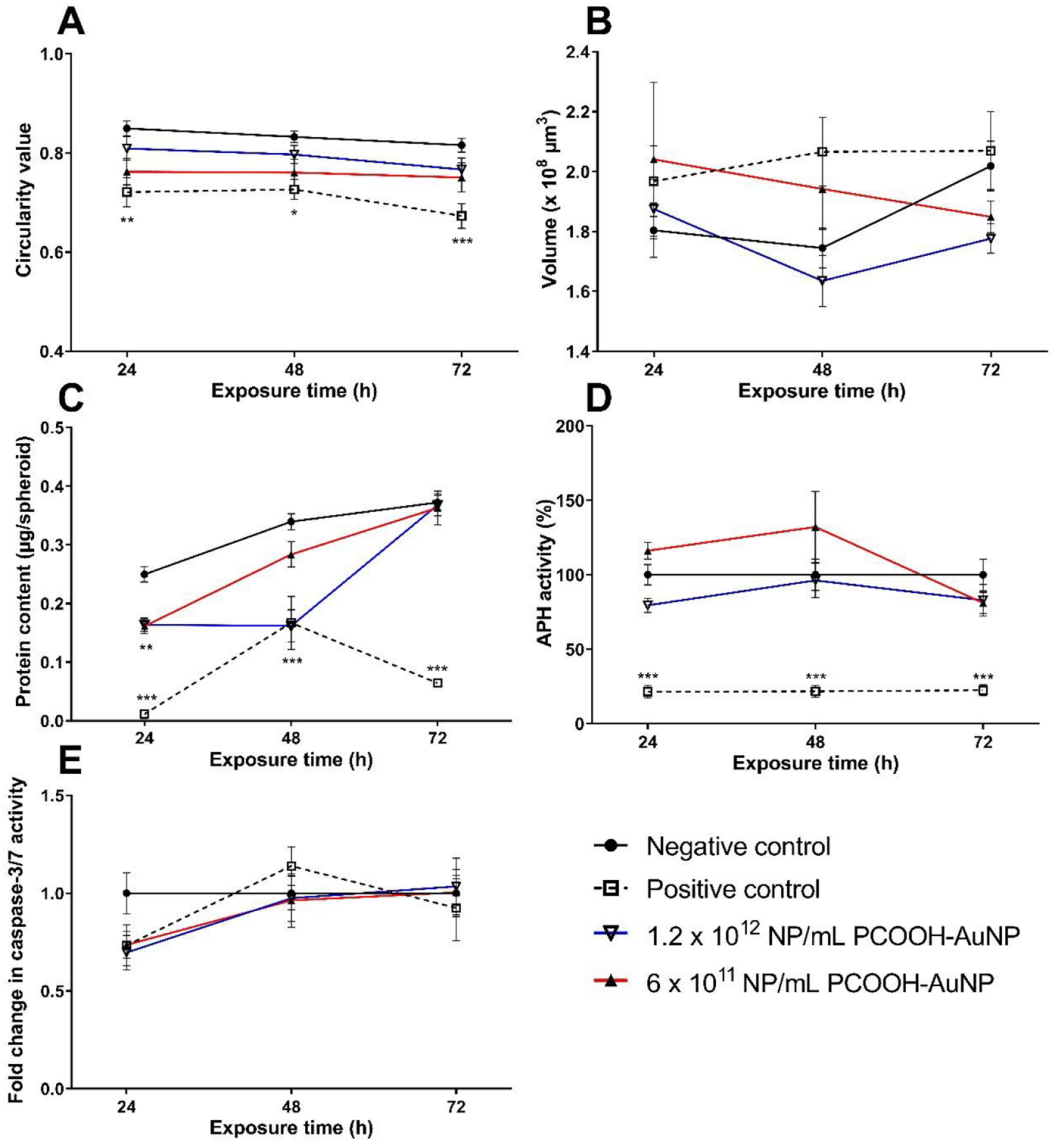
Effect of PCOOH‐liganded gold nanoparticles on the circularity index (A), volume (B), protein context (C), acid phosphatase activity (D), and caspase‐3/7 activity (E) of spheroids over a 72 h exposure period. Significance indicated as *p ≤ 0.05; **p ≤ 0.01; ***p ≤ 0.001 as compared to the negative control.

The PCOOH‐AuNPs non‐significantly (*p* > 0.05) decreased circularity (Figure 2A), suggesting minimal alterations at the concentrations tested. The PCOOH‐AuNPs at the lowest concentration tested (6 × 10^11^ NP/mL) non‐significantly increased spheroid volume after 48 h (11.34%) (Figure 2B), while the higher concentration of PCOOH‐AuNP (1.2 × 10^12^ NP/mL) did not (Figure 2B), though it is within the technical variation of one another.

The lower concentration of PCOOH‐AuNPs (6 × 10^11^ NP/mL) caused a significant (*p* < 0.05) decrease of 34.40% in protein content after 24 h, with this effect plateauing till 48 h, after which protein content reverted to baseline levels (Figure 2C). The higher concentration (1.2 × 10^12^ NP/mL) of PCOOH‐AuNPs resulted in a significant decrease (*p* < 0.01) in protein content of 35.45%, which diminished over time to become comparable to the negative control after 72 h (Figure 2C).

The 6 × 10^11^ NP/mL PCOOH‐AuNPs decreased APH activity non‐significantly by 20.57% between 24 and 48 h; however, this reverted to baseline by 72 h (Figure 2D). The higher concentration of PCOOH‐AuNPs (1.2 × 10^12^ NP/mL) induced the same trend in APH activity, with an initial increase from 24 to 48 h (16.14%, non‐significant), followed by a return to baseline after 72 h (Figure 2D).

**FIGURE 3:**
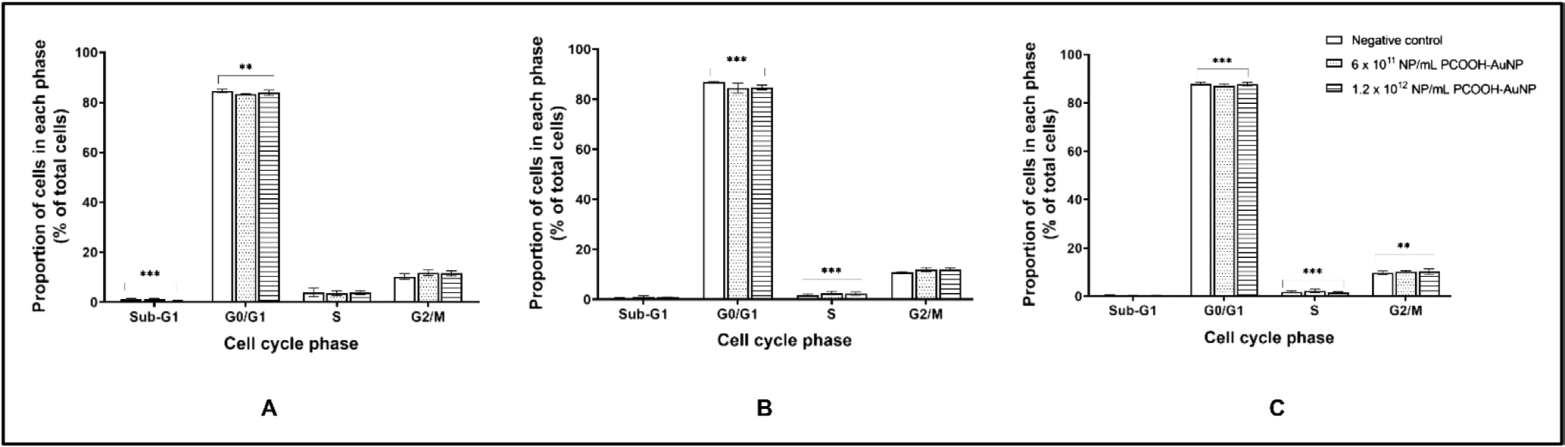
Effect of PCOOH‐liganded gold nanoparticle treatment on the distribution of A549 cells within the cell cycle of Day 7 A549 spheroids after 24 h (A), 48 h (B) and 72 h (C). Significance indicated as **p ≤ 0.01; ***p ≤ 0.001 as compared to the negative control.

After 24 h, the caspase‐3/7 activity was reduced, albeit non‐significantly (*p* > 0.05) for both concentrations of PCOOH‐AuNPs (Figure 2E). From 48 h onwards, the caspase‐3/7 activity returned to baseline for both concentrations of PCOOH‐AuNPs (Figure 2E). In comparison to the negative control, no statistically significant (*p* > 0.05) differences in cell cycle distribution were observed for either the 6 × 10^11^ NP/mL or 1.2 × 10^12^ NP/mL PCOOH‐AuNPs. The majority of cells (83.37%) were in the G_0_/G_1_ phase, with approximately 8% cycling from S to G_2_/M phase over the 72‐h exposure period (Figure 3).

#### 3.2.2 Amine‐Polyethylene Glycol (NH_2_ )‐Liganded AuNPs

**FIGURE 4:**
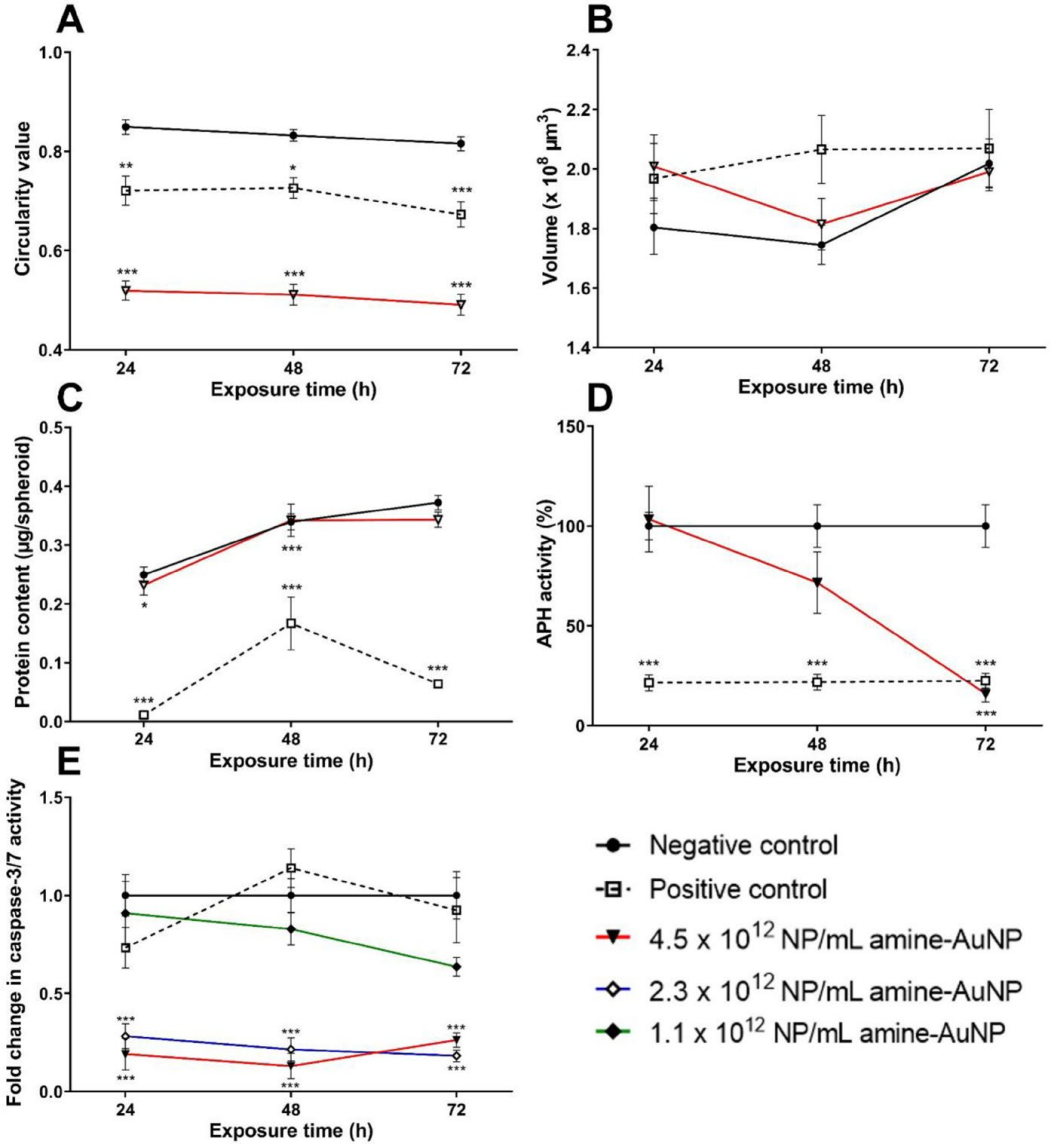
Effect of PNH_2_‐gold nanoparticles on the circularity index (A), volume (B), protein context (C), acid phosphatase activity (D), and caspase‐3/7 activity (E) of spheroids over a 72 h exposure period. Significance indicated as *p ≤ 0.05; **p ≤ 0.01; ***p ≤ 0.001 as compared to the negative control.

The PNH_2_‐AuNPs significantly (*p* < 0.001) decreased circularity by 38.84% after 24 h (Figure 4A) and then plateaued, suggesting a loss of shape and structural integrity. The PNH_2_‐AuNPs increased in volume by 10.24% after 24 h. The effect was similar to that of the positive control (Figure 4B), except that the effect occurred after a shorter exposure period.

The PNH_2_‐AuNPs decreased protein content non‐significantly (*p* > 0.05) at 72 h by 34.63% (Figure 4C). The APH activity of the 4.5 × 10^12^ NP/mL PNH_2_‐AuNPs‐exposed spheroids after 24 h was similar to the negative control (Figure 4D). A non‐significant (*p* > 0.05) decrease of 28.34% in APH activity was observed after 48 h, which was noted up to 72 h (83.99%; *p* < 0.001) (Figure 4D).

The lowest concentration (1.1 × 10^12^ NP/mL) of PNH_2_‐AuNPs caused a non‐significant (*p* > 0.05) decrease in caspase‐3/7 activity over the first 48 h; however, a significant reduction (*p* < 0.05) of 0.37‐fold was observed after 72 h (Figure 4E), compared to the negative control. The higher concentrations (2.3 × 10^12^ NP/mL and 4.5 × 10^12^ NP/mL) of PNH_2_‐AuNPs caused a significant decrease (*p* < 0.001) of 0.82 and 0.74‐fold, respectively, in caspase‐3/7 activity over 72 h, compared to the negative control (Figure 4E).

**FIGURE 5:**
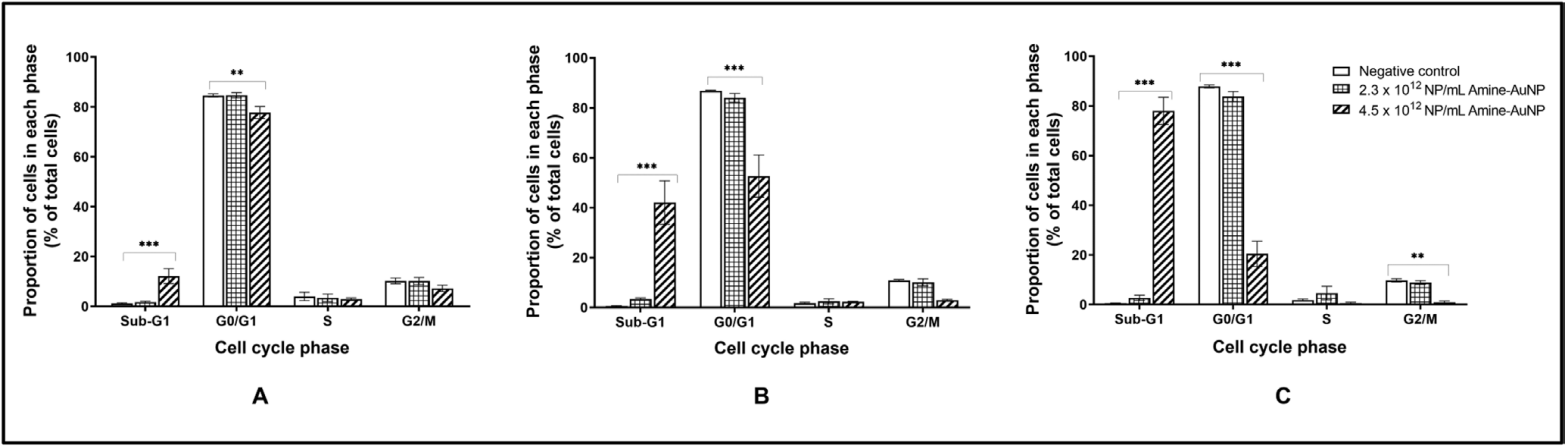
Effect of PNH_2_‐AuNPs treatment on the distribution of A549 cells within the cell cycle of Day 7 A549 spheroids after 24 h (A), 48 h (B), and 72 h (C). Significance indicated as **p ≤ 0.01; ***p ≤ 0.001, as compared to the negative control.

In comparison to the negative control, no statistically significant (*p* > 0.05) differences in cell cycle kinetics were observed after incubation with 2.3 × 10^12^ NP/mL or 1.2 × 10^12^ NP/mL PNH_2_‐AuNPs. The majority of cells (84.68%) were in the G_0_/G_1_ phase, with a low percentage cycling from G_2_/M to S‐phase over the 72‐h exposure period (Figure 5). However, the PNH_2_‐AuNPs at 4.5 × 10^12^ NP/mL resulted in a time‐dependent and significant (*p* < 0.001) increase in cells in the sub‐G_1_ phase, with a steady reduction in cells in the G_0_/G_1_ (significant; *p* < 0.01), S (non‐significant; *p* > 0.05), and G_2_/M phases (only significant at the 72 h time point; *p* < 0.01) from 24 h onwards (Figure 5). The proportion of cells in both the sub‐G_1_ and G_0_/G_1_ phases was significantly affected by the 4.5 × 10^12^ NP/mL PNH_2_‐AuNPs. After 24 h, a significant (*p* < 0.001) increase in the sub‐G_1_ phase and decrease (*p* < 0.01) in the G_0_/G_1_phase was noted. A similar trend was noted in the phases after 48 h. Ratiometrically, the percentage of cells relative to the G_0_/G_1_ phase of each respective time point suggests that cells are not cycling into the S and G_2_/M‐phase, and thus the ratio of cells has increased in the G_0_/G_1_‐phase, except at the 72 h time point where the 4.5 × 10^12^ NP/mL PNH_2_‐AuNPs produced the same effect with a significant increase in cells in the sub‐G_1_ phase (*p* < 0.001) and decrease in G_0_/G_1_‐phase (*p* < 0.001), while at the same time causing a significant a decrease in cells in the G_2_/M‐phase (*p* < 0.01).

## 4 Discussion

### 4.1 Establishing a 3D Model for A549 Spheroids

To facilitate reproducible high‐throughput assessment, homogenous spheroids are required for biological assays [38]. Although an adapted liquid overlay method [34] was used, the spheroids formed were often heterogeneous in shape and size. To circumvent this, a modified bulk generation liquid overlay method, using polydimethylsiloxane molds, was employed to generate spheroids, whereby self‐assembly occurs due to gravity in a low‐attachment environment [39, 40, 41]. This method proved more time‐efficient and resulted in the formation of up to 81 homogenous spheroids per single agarose micro‐mold.

The A549 spheroids displayed a diameter > 500 μm, a size at which zonal differentiation and nutrient/waste gradients occur, similar to in vivo tumors [42, 43, 44]. The zonal differentiation better reflects the diffusion kinetics of tumors, leading to greater representation of in vivo tumors, albeit influenced by spheroid circularity [45]. Spheroid circularity increased between Day 4 and Day 7, plateaued until Day 14, and systematically reduced thereafter. The latter is indicative of a gradual loss of integrity due to the inherent nature of scaffold‐free formation [43]. Previous characterization by the authors reported that A549 spheroids grown using the classic liquid overlay method had a larger average diameter of 702 μm on Day 7 [34] in comparison to the micro‐mold method (556.32 μm). Although the size of spheroids would be expected to increase during growth [46], the volume of Day 7 spheroids was decreased. Necrotic cores, which are generally seen in spheroids > 500 μm, form due to differential gradients of oxygen, nutrients, and waste leading to the accumulation of detrimental metabolic byproducts and reduced availability of cellular resources [46]. Such zonal differentiation provides more representation of 3D models in cytotoxicological studies [32, 33]. Given the increased APH activity and protein content, in parallel to the decreased volume, spheroid compaction is more likely due to increased cell–cell interactions [40, 43, 47].

### 4.2 The Effect of PCOOH‐Liganded Gold Nanoparticles on A549 Spheroids

The PCOOH‐AuNPs lacked cytotoxicity in all assessed parameters, which is supported by Fobian et al. using the lactate dehydrogenase assay [34]. Vetten and Gulumian also noted a lack of cytotoxicity after treatment with PCOOH‐AuNPs in monolayer cultures of the BEAS‐2B bronchial epithelial cell line [31]. PEGylated NPs have been reported to induce less cytotoxicity compared to their non‐PEGylated counterparts [17]. Given the low penetration of PCOOH‐AuNPs in A549 spheroids (regardless of the potential for clathrin‐mediated endocytosis leading to lysosomal trafficking) [34], it appears that the AuNPs may lack the means to alter intracellular processes or affect the inner structure of the spheroid. The lack of cytotoxicity would increase the attractiveness of the AuNPs as chemotherapeutic drug delivery agents; however, the minimal penetration via transcytosis perturbs its use [34].

The lack of alteration to cell viability is supported by Fobian et al. [34] and Vetten and Gulumian [31] The lower concentration of PCOOH‐AuNPs did affect protein content within the first 48 h, which may be indicative of protein degradation or loss of cells [48, 49], however, this did not translate to cytotoxicity. Covering the NP surface in PEG has been found to reduce protein deposition [50, 51]. The return of protein content to baseline implies that the effect could not be sustained after 48 h of exposure.

### 4.3 The Effect of PNH_2_ ‐Liganded Gold Nanoparticle on A549 Spheroids

The PNH_2_‐AuNPs were more cytotoxic than PCOOH‐liganded AuNPs, though these AuNPs did not affect protein content. The circularity index of spheroids was reduced significantly by PNH_2_‐AuNPs, indicating cytotoxicity and a loss of integrity. Although cellular proliferation in the periphery may also reduce rigid circularity due to newly formed cells lacking dense compaction [52], from the morphology a loss of structural integrity was noted. The uptake mechanism of the PNH_2_‐AuNPs is unknown, which warrants further investigation. Surface charge influences opsonization, which in turn affects plasma protein adsorption [53]. The PNH_2_‐AuNPs carry a slightly positive charge, with cationic liposomes known to have higher activation than neutral counterparts [54]. Harush‐Frenkel et al. found positively charged NPs to internalize more rapidly [55]. The slightly cationic nature of the PNH_2_‐AuNPs might explain the increased cytotoxicity observed, due to possible increased uptake compared to the PCOOH‐AuNPs.

The effect of the PNH_2_‐AuNPs on spheroid morphology increased after 48 h, suggesting further cell death, such as apoptosis, which is a time‐dependent mechanism [56]. When apoptosis is initiated, cell shrinkage occurs, and thus the overall volume of the spheroid decreases [56]. Brϋningk et al. found that reduced spheroid volume coincided with reduced viability in HCT116 cells [57]. The observed time‐dependence of the PNH_2_‐AuNPs's effects may be a factor of the uptake mechanism employed, NP design and penetration, and activation of cytotoxic mechanisms [58, 59, 60]. Since caspase‐3/7 activity was reduced, pro‐apoptotic caspase‐dependent activity was not present, and thus cytotoxicity may be driven by alternative pathways not assessed in the study. Cell death may be induced via caspase‐independent pathways, which do not require the activation of caspase enzymes [61, 62]. Lui et al. found that AuNPs (13 nm) induced cell death by various mechanisms, including caspase‐independent mechanisms [63]. Since spheroids are cultured to have contact with other cells and form an ECM, the spheroid may benefit from protection from anoikis (apoptosis incurred by loss of attachment to the extracellular matrix) [64, 65]. Loss of cellular or extracellular matrix contact (such as suggested by microscopy in the degradation of the spheroid structure) may have induced anoikis and subsequently apoptosis [66].

The PNH_2_‐AuNPs displayed dose‐dependent cell cycle disturbances, where only the highest concentration resulted in perturbations. This effect was also time‐dependent, which may be due to the gradual inhibition of cell cycle checkpoint factors [67]. Interestingly, the proportion of cells in the sub‐G_1_ phase increased after exposure to the PNH_2_‐AuNPs at the highest concentration tested, suggesting DNA fragmentation, which supports the potential for apoptosis. An increased proportion of cells in the sub‐G_1_ phase has been linked to DNA damage, protein degradation, and cytotoxicity caused by AuNPs [68]. Various AuNPs have been described to inflict DNA fragmentation [69, 70]. Ratiometrically, it appears that cells were not cycling past the G_0_/G_1_ phase for the first 48 h; however, at 72 h, the 4.5 × 10^12^ NP/mL PNH_2_‐AuNPs incurred DNA fragmentation and a block in the G_2_/M phase.

## 5 Conclusion

The spheroid model for A549 cells was successfully established and optimized via a high‐throughput micro‐mold system. The spheroids formed and started compacting by Day 4 and were sustained until Day 21. The spheroids remained stable over the three‐day exposure period, allowing for cytotoxicity determination. The PCOOH‐AuNPs did not have any significant cytotoxic effect on A549 spheroids. Although this finding appears to make them attractive targets for drug delivery vehicles, their uptake and cellular penetration are too low, as previously shown. PNH_2_‐AuNPs at high concentrations did significantly affect viability and growth measurements, indicating possible cytotoxicity. There is potential for future studies to establish whether the A549 spheroid would employ a different uptake mechanism compared to the findings on the PCOOH‐AuNPs. To further elucidate the extent and mechanism of DNA damage observed because of exposure to PNH_2_‐AuNPs, studies relating to anoikis and alternative mechanisms of cell death should be performed. The study has shown that the A549 spheroids established could reliably differentiate between non‐cytotoxic and cytotoxic AuNPs and thus serve as a platform for further development for drug discovery purposes.

## Author Contributions

M.P. performed all experimentation, contributed to the conceptualization of the project, analyzed all data, and drafted the manuscript. S‐.F.F. aided throughout with the development of the spheroid model and edited the manuscript. V.S. provided scientific insight and edited the manuscript. M.G. provided scientific insight on nanoparticles, funding, and nanoparticle supply and edited the manuscript. W.C. conceptualized the project, provided scientific insight and assistance with data analysis, and edited the manuscript.

## Ethics Statement

The project received ethical clearance from the Faculty of Health Sciences Research Ethics Committee of the University of Pretoria (690/2019).

## Consent

It is hereby confirmed that all authors are aware of the contents of this manuscript and provide consent for its publication.

## Conflicts of Interest

The authors declare no conflicts of interest.

## Supporting information


Data S1.

## Data Availability

Data are available upon request.

## References

[1] J. A. Barta , C. A. Powell , and J. P. Wisnivesky , “Global Epidemiology of Lung Cancer,” Annals of Globalization and Health 85, no. 1 (2019): 8. 10.5334/aogh.2419 PMC6724220 30741509

[2] R. L. Siegel , K. D. Miller , N. S. Wagle , and A. Jemal , “Cancer Statistics, 2023,” CA: A Cancer Journal for Clinicians 73, no. 1 (2023): 17–48. 36633525 10.3322/caac.21763

[3] Statistics South Africa , “Cancer in South Africa (2008–2019),” 2023, https://www.statssa.gov.za/publications/03‐08‐00/03‐08‐002023.pdf.

[4] E. Caliman , S. Fancelli , G. Petroni , et al., “Challenges in the Treatment of Small Cell Lung Cancer in the Era of Immunotherapy and Molecular Classification,” Lung Cancer 175 (2023): 88–100. 36493578 10.1016/j.lungcan.2022.11.014

[5] T. Sethi , R. C. Rintoul , S. M. Moore , et al., “Extracellular Matrix Proteins Protect Small Cell Lung Cancer Cells Against Apoptosis: A Mechanism for Small Cell Lung Cancer Growth and Drug Resistance In Vivo,” Nature Medicine 5, no. 6 (1999): 662–668. 10.1038/9511 10371505

[6] P. J. Morin , “Drug Resistance and the Microenvironment: Nature and Nurture,” Drug Resistance Updates 6, no. 4 (2003): 169–172. 12962682 10.1016/s1368-7646(03)00059-1

[7] M. M. Gottesman , “Mechanisms of Cancer Drug Resistance,” Annual Review of Medicine 53 (2002): 615–627. 10.1146/annurev.med.53.082901.103929 11818492

[8] J. Antunes , V. M. Gaspar , L. Ferreira , et al., “In‐Air Production of 3D Co‐Culture Tumor Spheroid Hydrogels for Expedited Drug Screening,” Acta Biomaterialia 94 (2019): 392–409. 31200118 10.1016/j.actbio.2019.06.012

[9] P. S. Hodkinson , A. C. Mackinnon , and T. Sethi , “Extracellular Matrix Regulation of Drug Resistance in Small‐Cell Lung Cancer,” International Journal of Radiation Biology 83, no. 11–12 (2007): 733–741. 17852559 10.1080/09553000701570204

[10] G. H. Williams and K. Stoeber , “The Cell Cycle and Cancer,” Journal of Pathology 226, no. 2 (2012): 352–364. 21990031 10.1002/path.3022

[11] D. Loessner , K. S. Stok , M. P. Lutolf , D. W. Hutmacher , J. A. Clements , and S. C. Rizzi , “Bioengineered 3D Platform to Explore Cell‐ECM Interactions and Drug Resistance of Epithelial Ovarian Cancer Cells,” Biomaterials 31, no. 32 (2010): 8494–8506. 20709389 10.1016/j.biomaterials.2010.07.064

[12] W. H. De Jong and P. J. A. Borm , “Drug Delivery and Nanoparticles: Applications and Hazards,” International Journal of Nanomedicine 3, no. 2 (2008): 133–149. 18686775 10.2147/ijn.s596 PMC2527668

[13] E. J. Cathcart‐Rake , L. R. Sangaralingham , H. J. Henk , N. D. Shah , I. B. Riaz , and A. S. Mansfield , “A Population‐Based Study of Immunotherapy‐Related Toxicities in Lung Cancer,” Clinical Lung Cancer 21, no. 5 (2020): 421–427. 32446852 10.1016/j.cllc.2020.04.003 PMC7486993

[14] J. Conde , G. Doria , and P. Baptista , “Noble Metal Nanoparticles Applications in Cancer,” Journal of Drug Delivery 2012 (2012): 1–12. 10.1155/2012/751075 PMC3189598 22007307

[15] A. D. Friedman , S. E. Claypool , and R. Liu , “The Smart Targeting of Nanoparticles,” Current Pharmaceutical Design 19, no. 35 (2013): 6315–6329. 23470005 10.2174/13816128113199990375 PMC4016770

[16] P. Ghosh , G. Han , M. De , C. K. Kim , and V. M. Rotello , “Gold Nanoparticles in Delivery Applications,” Advanced Drug Delivery Reviews 60, no. 11 (2008): 1307–1315. 18555555 10.1016/j.addr.2008.03.016

[17] R. Thiruppathi , S. Mishra , M. Ganapathy , P. Padmanabhan , and B. Gulyás , “Nanoparticle Functionalization and Its Potentials for Molecular Imaging,” Advanced Science 4, no. 3 (2017): 1600279. 28331783 10.1002/advs.201600279 PMC5357986

[18] C. M. Goodman , C. D. McCusker , T. Yilmaz , and V. M. Rotello , “Toxicity of Gold Nanoparticles Functionalized With Cationic and Anionic Side Chains,” Bioconjugate Chemistry 15, no. 4 (2004): 897–900. 15264879 10.1021/bc049951i

[19] S. L. Montes‐Fonseca , E. Orrantia‐Borunda , A. Aguilar‐Elguezabal , C. González Horta , P. Talamás‐Rohana , and B. Sánchez‐Ramírez , “Cytotoxicity of Functionalized Carbon Nanotubes in J774A Macrophages,” Nanomedicine: Nanotechnology, Biology and Medicine 8, no. 6 (2012): 853–859. 22033080 10.1016/j.nano.2011.10.002

[20] A. A. Shvedova , V. Castranova , E. R. Kisin , et al., “Exposure to Carbon Nanotube Material: Assessment of Nanotube Cytotoxicity Using Human Keratinocyte Cells,” Journal of Toxicology and Environmental Health 66, no. 20 (2003): 1909–1926. 14514433 10.1080/713853956

[21] D. M. Brown , K. Donaldson , P. J. Borm , et al., “Calcium and ROS‐Mediated Activation of Transcription Factors and TNF‐Alpha Cytokine Gene Expression in Macrophages Exposed to Ultrafine Particles,” American Journal of Physiology 286, no. 2 (2004): 344–353. 10.1152/ajplung.00139.2003 14555462

[22] R. A. Sperling , P. Rivera Gil , F. Zhang , M. Zanella , and W. J. Parak , “Biological Applications of Gold Nanoparticles,” Chemical Society Reviews 37, no. 9 (2008): 1896–1908. 18762838 10.1039/b712170a

[23] P. P. Edwards and J. M. Thomas , “Gold in a Metallic Divided State—From Faraday to Present‐Day Nanoscience,” Angewandte Chemie 46, no. 29 (2007): 5480–5486. 17562538 10.1002/anie.200700428

[24] M. Das , K. H. Shim , S. S. A. An , and D. K. Yi , “Review on Gold Nanoparticles and Their Applications,” Toxicology and Environmental Health Sciences 3, no. 4 (2011): 193–205.

[25] K. Saha , S. S. Agasti , C. Kim , X. Li , and V. M. Rotello , “Gold Nanoparticles in Chemical and Biological Sensing,” Chemical Reviews 112, no. 5 (2012): 2739–2779. 22295941 10.1021/cr2001178 PMC4102386

[26] L. Torrisi , “Gold Nanoparticles Enhancing Proton Therapy Efficiency,” Recent Patents on Nanotechnology 9, no. 1 (2015): 51–60. 25986229 10.2174/1872210509999141222224121

[27] B. D. Chithrani , A. A. Ghazani , and W. C. Chan , “Determining the Size and Shape Dependence of Gold Nanoparticle Uptake Into Mammalian Cells,” Nano Letters 6, no. 4 (2006): 662–668. 16608261 10.1021/nl052396o

[28] E. E. Connor , J. Mwamuka , A. Gole , C. J. Murphy , and M. D. Wyatt , “Gold Nanoparticles Are Taken up by Human Cells but Do Not Cause Acute Cytotoxicity,” Small 1, no. 3 (2005): 325–327. 17193451 10.1002/smll.200400093

[29] H. K. M. Patra , S. M. Banerjee , U. M. D. Chaudhuri , P. P. Lahiri , and A. K. P. Dasgupta , “Cell Selective Response to Gold Nanoparticles,” Nanomedicine: Nanotechnology, Biology and Medicine 3, no. 2 (2007): 111–119. 17572353 10.1016/j.nano.2007.03.005

[30] Y. Pan , A. Leifert , D. Ruau , et al., “Gold Nanoparticles of Diameter 1.4 nm Trigger Necrosis by Oxidative Stress and Mitochondrial Damage,” Small 5, no. 18 (2009): 2067–2076. 19642089 10.1002/smll.200900466

[31] M. Vetten and M. Gulumian , “Differences in Uptake of 14 nm PEG‐Liganded Gold Nanoparticles Into BEAS‐2B Cells Is Dependent on Their Functional Groups,” Toxicology and Applied Pharmacology 363 (2019): 131–141. 30529166 10.1016/j.taap.2018.11.014

[32] X. Yan , H. Luo , X. Zhou , B. Zhu , Y. Wang , and X. Bian , “Identification of CD90 as a Marker for Lung Cancer Stem Cells in A549 and H446 Cell Lines,” Oncology Reports 30, no. 6 (2013): 2733–2740. 24101104 10.3892/or.2013.2784

[33] F.‐F. Sun , Y.‐H. Hu , L.‐P. Xiong , et al., “Enhanced Expression of Stem Cell Markers and Drug Resistance in Sphere‐Forming Non‐Small Cell Lung Cancer Cells,” International Journal of Clinical and Experimental Pathology 8, no. 6 (2015): 6287–6300. 26261505 PMC4525839

[34] S.‐F. Fobian , M. Petzer , M. Vetten , V. Steenkamp , M. Gulumian , and W. Cordier , “Mechanisms Facilitating the Uptake of Carboxyl–Polythene Glycol‐Functionalized Gold Nanoparticles Into Multicellular Spheroids,” Journal of Pharmacy and Pharmacology 74 (2022): rgac017. 10.1093/jpp/rgac017 35417021

[35] G. Frens , “Controlled Nucleation For The Regulation Of The Particle Size in Monodisperse Gold Suspensions,” Nature Physical Science 241, no. 105 (1973): 20–22.

[36] J. Turkevich , P. C. Stevenson , and J. Hillier , “A Study of the Nucleation and Growth Processes in the Synthesis of Colloidal Gold,” Discussions of the Faraday Society 11 (1951): 55–75.

[37] D. Hammill , “Cytoexplorer: Interactive Analysis of Cytometry Data 2021,” 2023, https://dillonhammill.github.io/CytoExploreR/index.html.

[38] T.‐M. Achilli , J. Meyer , and J. R. Morgan , “Advances in the Formation, Use and Understanding of Multi‐Cellular Spheroids,” Expert Opinion on Biological Therapy 12, no. 10 (2012): 1347–1360. 22784238 10.1517/14712598.2012.707181 PMC4295205

[39] J. M. Kelm and M. Fussenegger , “Microscale Tissue Engineering Using Gravity‐Enforced Cell Assembly,” Trends in Biotechnology 22, no. 4 (2004): 195–202. 15038925 10.1016/j.tibtech.2004.02.002

[40] A. P. Napolitano , P. Chai , D. M. Dean , and J. R. Morgan , “Dynamics of the Self‐Assembly of Complex Cellular Aggregates on Micromolded Non‐Adhesive Hydrogels,” Tissue Engineering 13, no. 8 (2007): 2087–2094. 17518713 10.1089/ten.2006.0190

[41] L. P. Ferreira , V. M. Gaspar , and J. F. Mano , “Bioinstructive Microparticles for Self‐Assembly of Mesenchymal Stem Cell‐3D Tumor Spheroids,” Biomaterials 185 (2018): 155–173. 30245385 10.1016/j.biomaterials.2018.09.007 PMC7617209

[42] D. Khaitan , S. Chandna , M. B. Arya , and B. S. Dwarakanath , “Establishment and Characterization of Multicellular Spheroids From a Human Glioma Cell Line; Implications for Tumor Therapy,” Journal of Translational Medicine 4 (2006): 12. 16509995 10.1186/1479-5876-4-12 PMC1420330

[43] M. Zanoni , F. Piccinini , C. Arienti , et al., “3D Tumor Spheroid Models for In Vitro Therapeutic Screening: A Systematic Approach to Enhance the Biological Relevance of Data Obtained,” Scientific Reports 6, no. 1 (2016): 19103. 26752500 10.1038/srep19103 PMC4707510

[44] S. Nath and G. R. Devi , “Three‐Dimensional Culture Systems in Cancer Research: Focus on Tumor Spheroid Model,” Pharmacology & Therapeutics 163 (2016): 94–108. 27063403 10.1016/j.pharmthera.2016.03.013 PMC4961208

[45] B. M. Leung , S. C. Lesher‐Perez , T. Matsuoka , C. Moraes , and S. Takayama , “Media Additives to Promote Spheroid Circularity and Compactness in Hanging Drop Platform,” Biomaterials Science 3, no. 2 (2015): 336–344. 26218124 10.1039/c4bm00319e

[46] H. Lu and M. H. Stenzel , “Multicellular Tumor Spheroids (MCTs) as an 3D in Vitro Evaluation Tool of Nanoparticles,” Small 14, no. 13 (2018): 1702858. 10.1002/smll.201702858 29450963

[47] R.‐Z. Lin , L.‐F. Chou , C.‐C. M. Chien , and H.‐Y. Chang , “Dynamic Analysis of Hepatoma Spheroid Formation: Roles of E‐Cadherin and Β1‐Integrin,” Cell and Tissue Research 324, no. 3 (2006): 411–422. 16489443 10.1007/s00441-005-0148-2

[48] M. Kus‐Liśkiewicz , P. Fickers , and T. I. Ben , “Biocompatibility and Cytotoxicity of Gold Nanoparticles: Recent Advances in Methodologies and Regulations,” International Journal of Molecular Sciences 22, no. 20 (2021): 10952. 34681612 10.3390/ijms222010952 PMC8536023

[49] J. K. Tee , C. N. Ong , B. H. Bay , H. K. Ho , and D. T. Leong , “Oxidative Stress by Inorganic Nanoparticles,” WIREs Nanomedicine and Nanobiotechnology 8, no. 3 (2016): 414–438. 26359790 10.1002/wnan.1374

[50] I. Lynch , T. Cedervall , M. Lundqvist , C. Cabaleiro‐Lago , S. Linse , and K. A. Dawson , “The Nanoparticle‐Protein Complex as a Biological Entity; a Complex Fluids and Surface Science Challenge for the 21st Century,” Advances in Colloid and Interface Science 134‐135 (2007): 167–174. 10.1016/j.cis.2007.04.021 17574200

[51] W. Norde and R. A. Gage , “Interaction of Bovine Serum Albumin and Human Blood Plasma With PEO‐Tethered Surfaces: Influence of PEO Chain Length, Grafting Density and Temperature,” Langmuir 20, no. 10 (2004): 4162–4167. 15969411 10.1021/la030417t

[52] X. Gong , C. Lin , J. Cheng , et al., “Generation of Multicellular Tumor Spheroids With Microwell‐Based Agarose Scaffolds for Drug Testing,” PLoS One 10, no. 6 (2015): e0130348. 26090664 10.1371/journal.pone.0130348 PMC4474551

[53] Z. Zhao , A. Ukidve , V. Krishnan , and S. Mitragotri , “Effect of Physicochemical and Surface Properties on In Vivo Fate of Drug Nanocarriers,” Advanced Drug Delivery Reviews 143 (2019): 3–21. 30639257 10.1016/j.addr.2019.01.002

[54] S. C. Semple , A. Chonn , and P. R. Cullis , “Interactions of Liposomes and Lipid‐Based Carrier Systems With Blood Proteins: Relation to Clearance Behaviour In Vivo,” Advanced Drug Delivery Reviews 32, no. 1 (1998): 3–17. 10837632 10.1016/s0169-409x(97)00128-2

[55] O. Harush‐Frenkel , N. Debotton , S. Benita , and Y. Altschuler , “Targeting of Nanoparticles to the Clathrin‐Mediated Endocytic Pathway,” Biochemical and Biophysical Research Communications 353, no. 1 (2007): 26–32. 17184736 10.1016/j.bbrc.2006.11.135

[56] D. P. Ivanov , T. L. Parker , D. A. Walker , et al., “Multiplexing Spheroid Volume, Resazurin and Acid Phosphatase Viability Assays for High‐Throughput Screening of Tumour Spheroids and Stem Cell Neurospheres,” PLoS One 9 (2014): e103817. 25119185 10.1371/journal.pone.0103817 PMC4131917

[57] S. C. Brüningk , I. Rivens , C. Box , U. Oelfke , and G. ter Haar , “3D Tumour Spheroids for the Prediction of the Effects of Radiation and Hyperthermia Treatments,” Scientific Reports 10, no. 1 (2020): 1653. 32015396 10.1038/s41598-020-58569-4 PMC6997397

[58] B. Salesa , M. Assis , J. Andrés , and Á. Serrano‐Aroca , “Carbon Nanofibers Versus Silver Nanoparticles: Time‐Dependent Cytotoxicity, Proliferation, and Gene Expression,” Biomedicine 9, no. 9 (2021): 1155. 10.3390/biomedicines9091155 PMC8467915 34572341

[59] M. Paknijadi , M. Bayat , M. Salimi , and V. Razavilar , “Concentration‐ and Time‐Dependent Cytotoxicity of Silver Nanoparticles on Normal Human Skin Fibroblast Cell Line,” Iranian Red Crescent Medical Journal 20, no. 10 (2024): 1–8.

[60] E. Efeoglu , A. Casey , and H. J. Byrne , “In Vitro Monitoring of Time and Dose Dependent Cytotoxicity of Aminated Nanoparticles Using Raman Spectroscopy,” Analyst 141, no. 18 (2016): 5417–5431. 27373561 10.1039/c6an01199c

[61] S. Kumar , “Caspase Function in Programmed Cell Death,” Cell Death and Differentiation 14, no. 1 (2007): 32–43. 17082813 10.1038/sj.cdd.4402060

[62] D. De Stefano , R. Carnuccio , and M. C. Maiuri , “Nanomaterials Toxicity and Cell Death Modalities,” Journal of Drug Delivery 14 (2012): 167896. 10.1155/2012/167896 PMC3523142 23304518

[63] M. Liu , X. Gu , K. Zhang , et al., “Gold Nanoparticles Trigger Apoptosis and Necrosis in Lung Cancer Cells With Low Intracellular Glutathione,” Journal of Nanoparticle Research 15, no. 8 (2013): 1745.

[64] P. Paoli , E. Giannoni , and P. Chiarugi , “Anoikis Molecular Pathways and Its Role in Cancer Progression,” Biochimica et Biophysica Acta (BBA)—Molecular Cell Research 1833, no. 12 (2013): 3481–3498. 23830918 10.1016/j.bbamcr.2013.06.026

[65] S. Malagobadan and N. H. Nagoor , “Anoikis,” in Encyclopedia of Cancer, 3rd ed., eds. P. Boffetta , and P. Hainaut (Oxford: Academic Press, 2019), 75–84.

[66] P. Chiarugi and E. Giannoni , “Anoikis: A Necessary Death Program for Anchorage‐Dependent Cells,” Biochemical Pharmacology 76, no. 11 (2008): 1352–1364. 18708031 10.1016/j.bcp.2008.07.023

[67] A. Panagopoulos and M. Altmeyer , “The Hammer and the Dance of Cell Cycle Control,” Trends in Biochemical Sciences 46, no. 4 (2021): 301–314. 33279370 10.1016/j.tibs.2020.11.002

[68] J. A. Coulter , S. Jain , K. T. Butterworth , et al., “Cell Type‐Dependent Uptake, Localization, and Cytotoxicity of 1.9 nm Gold Nanoparticles,” International Journal of Nanomedicine 7 (2012): 2673–2685. 22701316 10.2147/IJN.S31751 PMC3373299

[69] R. Geetha , T. Ashokkumar , S. Tamilselvan , K. Govindaraju , M. Sadiq , and G. Singaravelu , “Green Synthesis of Gold Nanoparticles and Their Anticancer Activity,” Cancer Nanotechnology 4, no. 4 (2013): 91–98. 26069504 10.1007/s12645-013-0040-9 PMC4451866

[70] H. Y. El‐Kassas and M. M. El‐Sheekh , “Cytotoxic Activity of Biosynthesized Gold Nanoparticles With an Extract of the Red Seaweed *Corallina officinalis* on the MCF‐7 Human Breast Cancer Cell Line,” Asian Pacific Journal of Cancer Prevention 15, no. 10 (2014): 4311–4317. 24935390 10.7314/apjcp.2014.15.10.4311

