# Supplementary material for: A549 Alveolar Carcinoma Spheroids as a Cytotoxicity Platform for Carboxyl‐ and Amine‐Polyethylene Glycol Gold Nanoparticles
Source: Pharmacol Res Perspect. 2024 Dec 26;13(1):e70051. doi: 10.1002/prp2.70051 (PMC11671224; doi:10.1002/prp2.70051)
Supplement: Supplementary file 1 — Data S1. [file PRP2-13-e70051-s001.docx]

## Supplementary File 1

## Nanoparticle interference studies with assay substrates

To determine whether the AuNPs would affect any of the experimental procedures, interference studies were conducted. Apart from the optical properties of the AuNPs, the potential to alter substrate conversion or fluorescence intensity of fluorophores was also assessed.

### Acid phosphatase assay

#### *Inherent effect on optical density*

The AuNPs displayed an increased absorbance when diluted in water (72.78% with 6 x 10^11^ NP/mL PCOOH-AuNP, 143.54% with 1.2 x 10^12^ NP/mL PCOOH-AuNP and 150.43% with 4.5 x 10^12^ NP/mL amine-AuNP), compared to only water (**Figure 1 A**). These increases in absorbance were non-significant (p > 0.05), for all but the 4.5 x 10^12^ NP/mL amine-AuNP group (p = 0.01), however the latter was considered negligible since the assay protocol does not include water.

A marginal increase in absorbance was observed when AuNPs were diluted in DMEM (7.8% for 6 x 10^11^ NP/mL PCOOH-AuNP, 21.32% with 1.2 x 10^12^ NP/mL PCOOH-AuNP and 41.17% with 4.5 x 10^12^ NP/mL amine-AuNP), compared to only DMEM (**Figure 1 A**). These increases in absorbance were non-significant (p > 0.05), for all but the 4.5 x 10^12^ NP/mL amine-AuNP group (p = 0.01). Although an increase was noted, it should be considered that in the cellular system, excess NPs that were not taken up by the cells are washed out of the external environment, thus decreasing their presence.

To indicate how marginal of an increase this was, the negative control in a cellular environment, where the assay is conducted in a medium-free environment, had an average absorbance value of 0.157, which is correlated to an increase of 220.41%, compared to water alone, and 15.44% compared to DMEM alone.

#### *Effect in the presence of assay substrates*

When comparing the observed absorbance of the AuNPs with the assay reagents (**Figure 1 B**), no significant spontaneous conversion of the substrate was observed (p > 0.05), except for the 1.2 x 10^12^ NP/mL PCOOH-AuNP, which showed an increased absorbance of 127.27%, compared to only the assay buffer. Although this result was significant (p < 0.05), it should be noted that in the experimental setting, wash steps will decrease the concentrations of the AuNPs, particularly as a low uptake into cells can be expected based on prior experimentation.


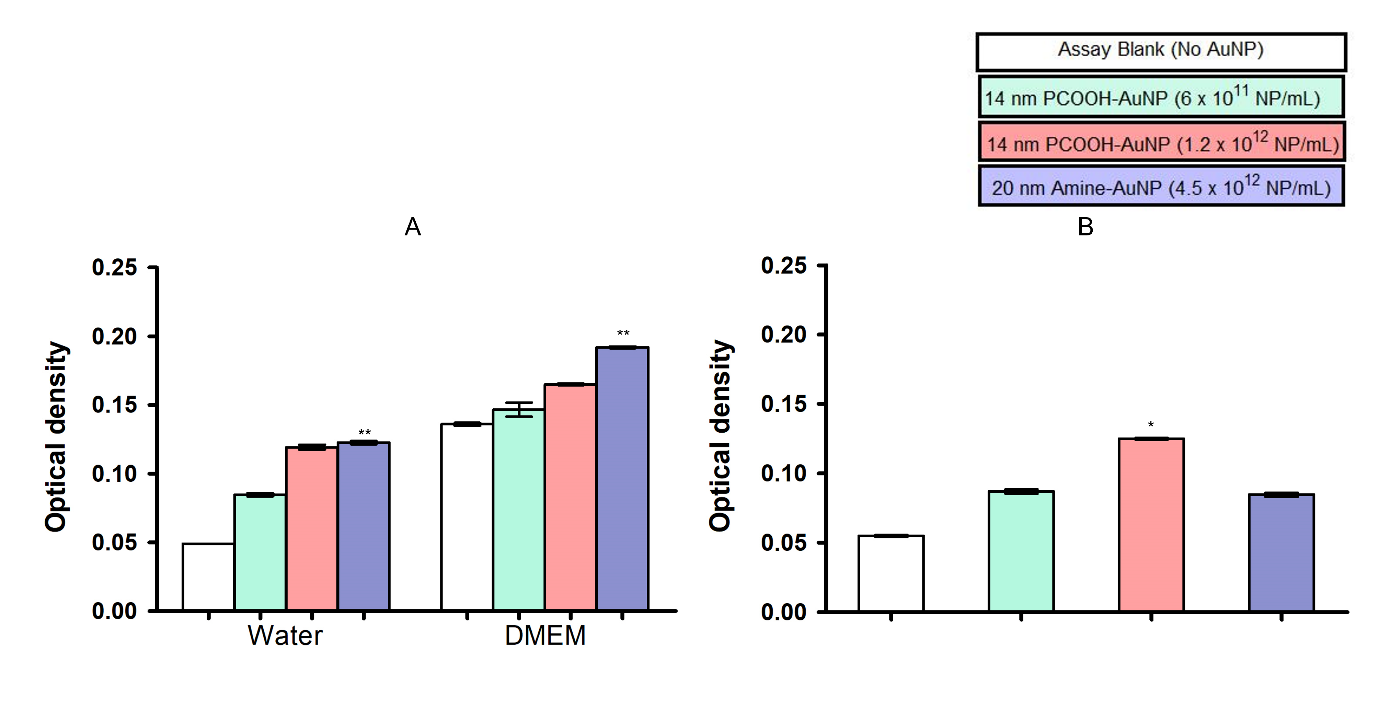


Figure 1: Absorbance values of gold nanoparticles in water and medium free from foetal calf serum (A) and interference of gold nanoparticles with the acid phosphatase assay substrate in a cell-free environment (B). Blank absorbance values were included for scale. The measurement was carried out at a wavelength of 405 nm. Significance indicated as *p ≤ 0.05; **p ≤ 0.01.

### Bicinchoninic acid assay

#### *Inherent effect on optical density*

The AuNPs displayed an increased absorbance when diluted in water (71.3% with 6 x 10^11^ NP/mL PCOOH-AuNP, 155.65% with 1.2 x 10^12^ NP/mL PCOOH-AuNP and 193.91% with 4.5 x 10^12^ NP/mL amine-AuNP), compared to that of water only (**Figure 2 A**). These increases in absorbance were non-significant (p > 0.05), for all but the 4.5 x 10^12^ NP/mL amine-AuNP group (p-value = 0.01), but was considered negligible since the assay protocol does not include water only. Although the assay protocol involves a number of wash steps, these were not included in the interference study due to the high probability that the AuNPs would be washed away.

The change in absorbance was marginal in the presence of AuNPs with DMEM. An absorbance increase of 0.53% with 6 x 10^11^ NP/mL PCOOH-AuNP, 12.82% decrease with 1.2 x 10^12^ NP/mL PCOOH-AuNP and 22.72% increase with 4.5 x 10^12^ NP/mL amine-AuNP was noted when compared to DMEM only (**Figure 2 A**). Changes in absorbance were non-significant (p > 0.05) for all three AuNPs and considered negligible due to the extensive washing involved in the assay protocol, which was not possible with the interference study due to the high probability of AuNPs being washed away, if not taken up by the cells.

#### *Effect in the presence of assay substrates*

When comparing the observed absorbance of the AuNPs with the assay reagents (**Figure 2 B**), no statistically significant (p > 0.05) spontaneous conversion of the substrate was observed, except for a 16.67% increase caused by the 4.5 x 10^12^ NP/mL amine-AuNPs when diluted in the buffer (p = 0.01). Although the assay protocol involves a number of wash steps, these were not included in the interference study due to the high probability that the AuNPs would be washed away and thus, this increase was considered non-relevant.


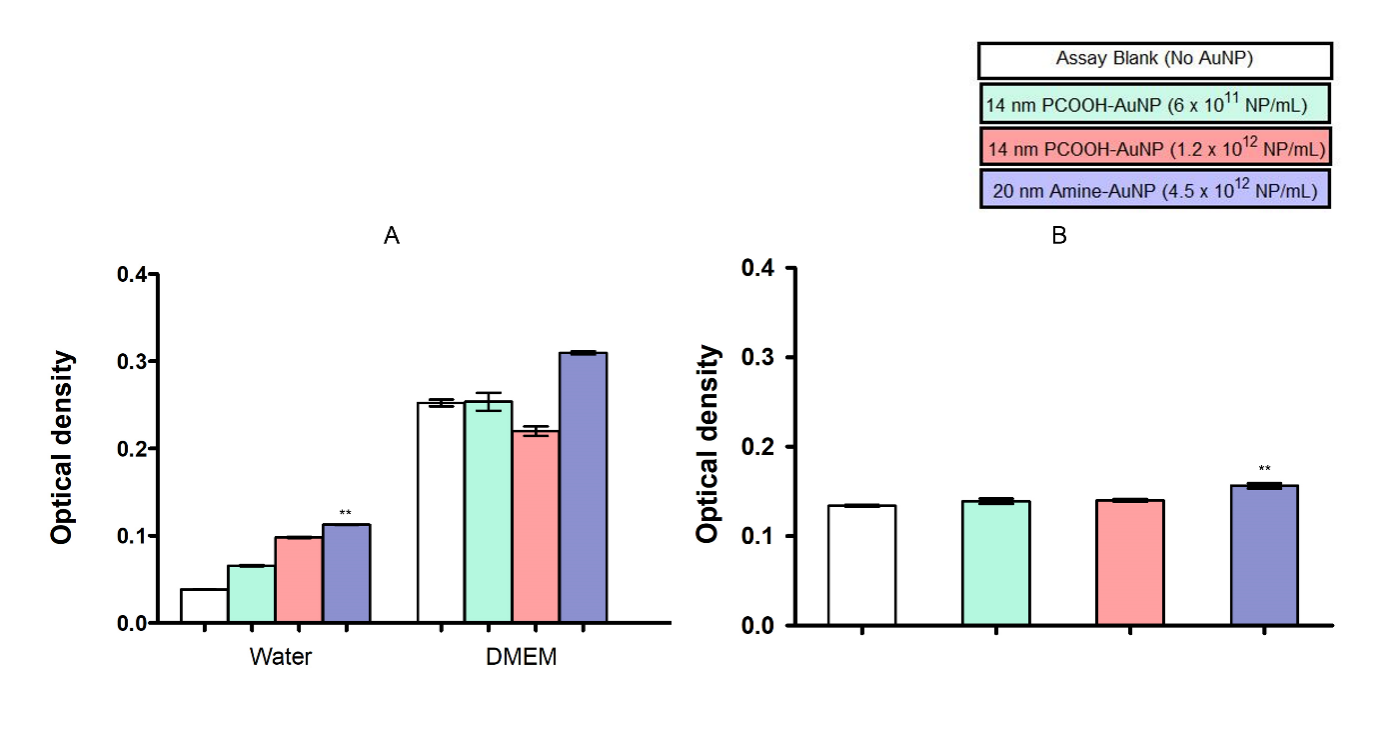


Figure 2: Absorbance values of gold nanoparticles in water and medium without foetal calf serum (A) and interference of gold nanoparticles with the acid phosphatase assay substrate in a cell-free environment (B). Blank absorbance values were included for scale. The measurement was carried out at a wavelength of 405 nm. Significance indicated as *p ≤ 0.05; **p ≤ 0.01.

### Propidium iodide fluorescence

#### *Autofluorescence of nanoparticles*

The AuNPs displayed a decrease in fluorescence when diluted in water (with 10.58% for 6 x 10^11^ NP/mL PCOOH-AuNP, 14.81% with 1.2 x 10^12^ NP/mL PCOOH-AuNP and 14.81% with 4.5 x 10^12^ NP/mL amine-AuNP), compared to water only (**Figure 3 A**). All decreases were non-significant (p > 0.05) and considered negligible except for the 4.5 x 10^12^ NP/mL amine-AuNPs, where there was a significant (p = 0.02) decrease in fluorescence, which was considered negligible due to the extensive washing that occurs during cellular assays.

When AuNPs were present in DMEM, fluorescence was decreased (with 20.35% for 6 x 10^11^ NP/mL PCOOH-AuNP, 39.63% with 1.2 x 10^12^ NP/mL PCOOH-AuNP and 12.94% with 4.5 x 10^12^ NP/mL amine-AuNP), compared to DMEM only (**Figure 3 A**). Although the fluorescent decrease observed with 1.2 x 10^12^ NP/mL PCOOH-AuNP was significant (p < 0.05), it was considered negligible due to the extensive washing that occurs during cellular assays.

#### *Possibility of propidium iodide fluorescent quenching by nanoparticles*

The AuNPs did not cause relevant quenching of PI, at either the low (2 mg/mL) or high (5 mg/mL) concentrations of PI (**Figure 3 B**). The marginal quenching effects observed were non-significant (p > 0.05), except for the quenching by 4.5 x 10^12^ NP/mL amine-AuNPs at 2 mg/mL PI (p < 0.05) and 1.2 x 10^12^ NP/mL PCOOH-AuNP at 5 mg/mL PI (p < 0.05). The effect was considered low enough for the analysis to continue, since the in-reaction concentration of PI in the protocol was much lower than the concentrations tested, as well as the extensive washing prior to taking the readings.


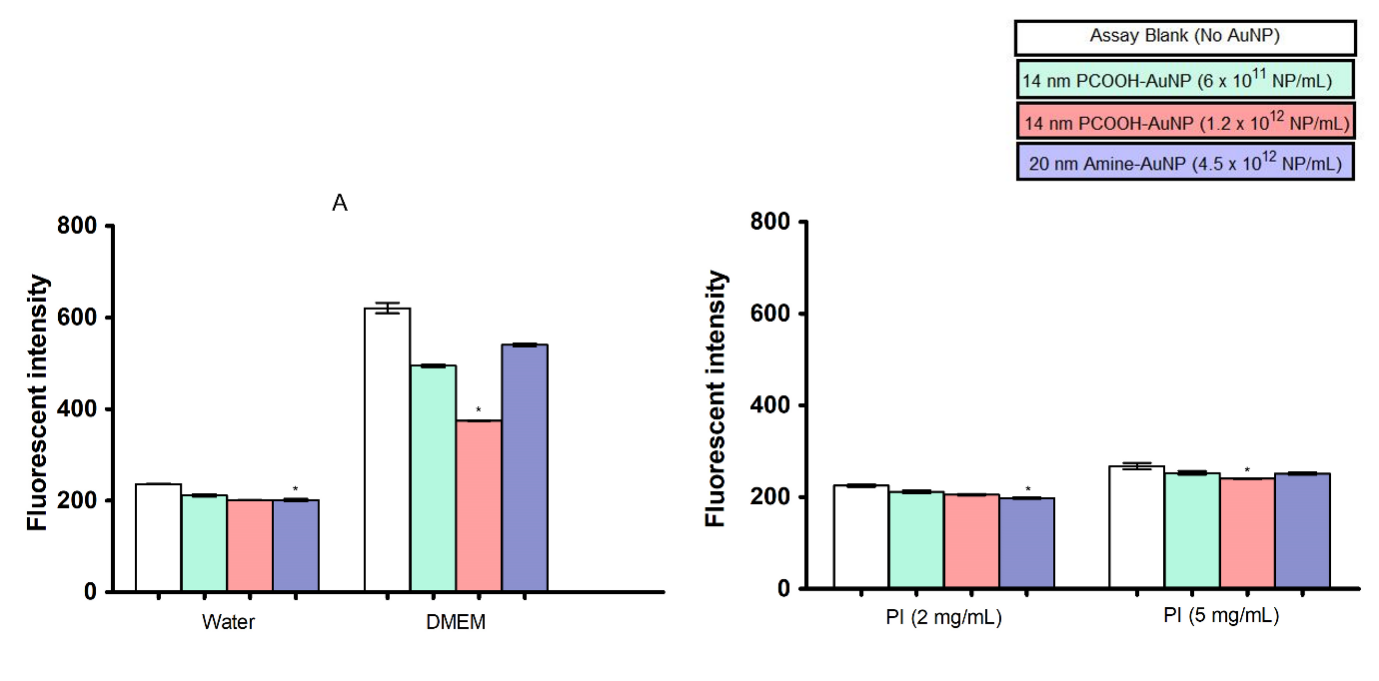


Figure 3: Fluorescent intensity of the gold nanoparticles in water and medium devoid of foetal calf serum (A) and quenching of propidium iodide (PI) at high concentration by gold nanoparticles (AuNP) (B). Blank values were included for reference. The 385±40, 590±35 filter set (nm; excitation, emission wavelengths ± bandpass filter ranges) was used. Significance indicated as *p ≤ 0.05.

#### *Possibility of nanoparticle identification during flow cytometry*

Interference studies were undertaken using the CytoFlex flow cytometer to determine whether the AuNPs would be picked up as fluorescent debris during flow cytometry (**Figures 4** and **5**) or interfere with PI fluorescence. The analysis was performed with AuNPs were at maximum concentrations; 1.2 x 10^12^ NP/mL PCOOH-AuNP (**Figure 4**) and 4.5 x 10^12^ NP/mL amine-AuNP (**Figure 5**) for 24 h. It was found that in the absence of cells (green and red graphs on **Figures 4** and **5**), the AuNPs were not picked up as being part of the cell cycle. Cell cycle activity was only observed in groups that did not contain any cells (yellow and blue on **Figures 4** and **5**). Propidium iodide concentrations were comparable to the experimental protocol.


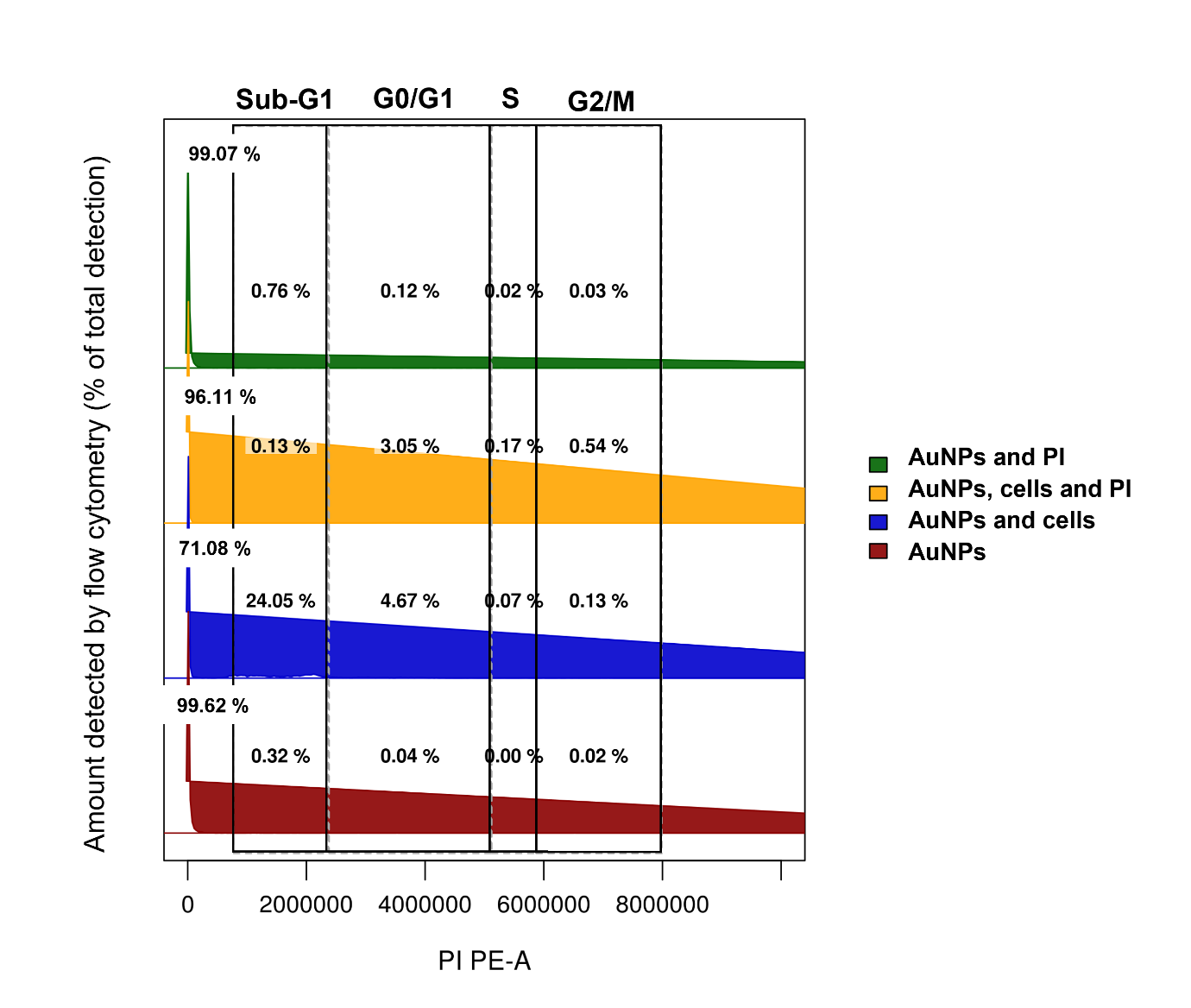


Figure 4: Percentage of material picked up by CytoFlex flow cytometer to determine whether PCOOH-AuNPs would interfere with propidium iodide (PI) during experimental analysis.


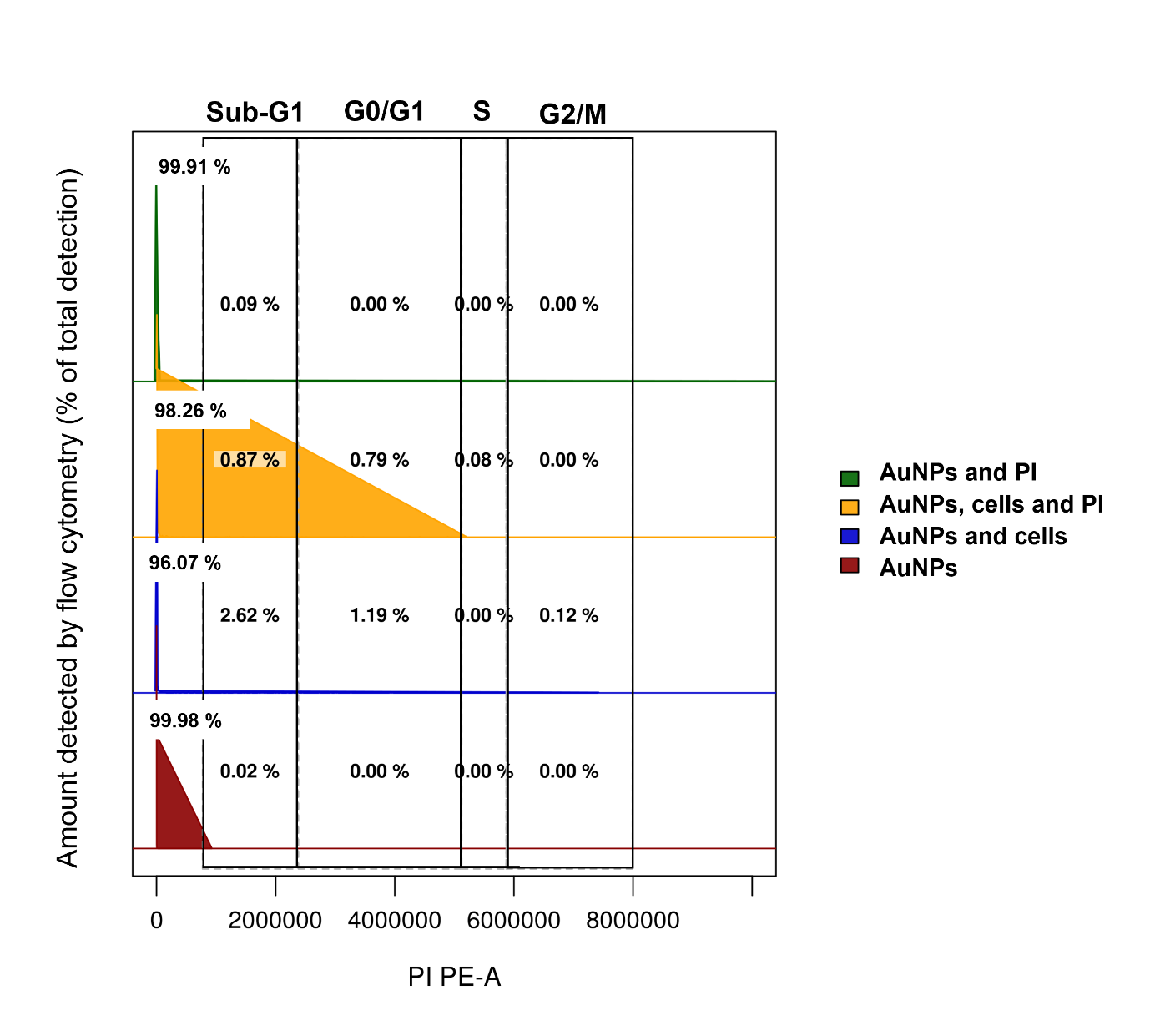


Figure 5: Percentage of material picked up by CytoFlex flow cytometer to determine whether amine-AuNPs would interfere with propidium iodide (PI) during experimental analysis.

### Caspase-3/7 assay

There was a non-significant decrease in fluorescence (p > 0.05), of 40.00% for 6 x 10^11^ NP/mL PCOOH-AuNP, 35.15% with 2.3 x 10^12^ NP/mL amine-AuNP and 49.99% with 4.5 x 10^12^ NP/mL amine-AuNP when diluted in water, compared to water only (**Figure 6**). There was a significant decrease (p < 0.01) decrease in fluorescence by 56.56% with 1.2 x 10^12^ NP/mL PCOOH-AuNP (**Figure 6**). Although it might have been of concern since the assay was performed in a water-based solution, it was considered negligible due to the extensive washing involved in the assay protocol, which was not possible with the interference study due to the high probability of AuNPs being washed away, if not taken up by the cells.

A non-significant (p > 0.05) decrease in fluorescence was noted for both AuNPs (low concentrations) when diluted in DMEM, compared to DMEM only (**Figure 6**). There were significant decreases in fluorescence (p < 0.05) for both groups (**Figure 6**) at high concentrations. The latter was considered negligible especially considering the number of wash-steps involved, implying that there would essentially be no DMEM present at the time at which the fluorescence was read. There was extensive washing involved in the assay protocol, which was not possible with the interference study due to the high probability of AuNPs being washed away, if not taken up by the cells.


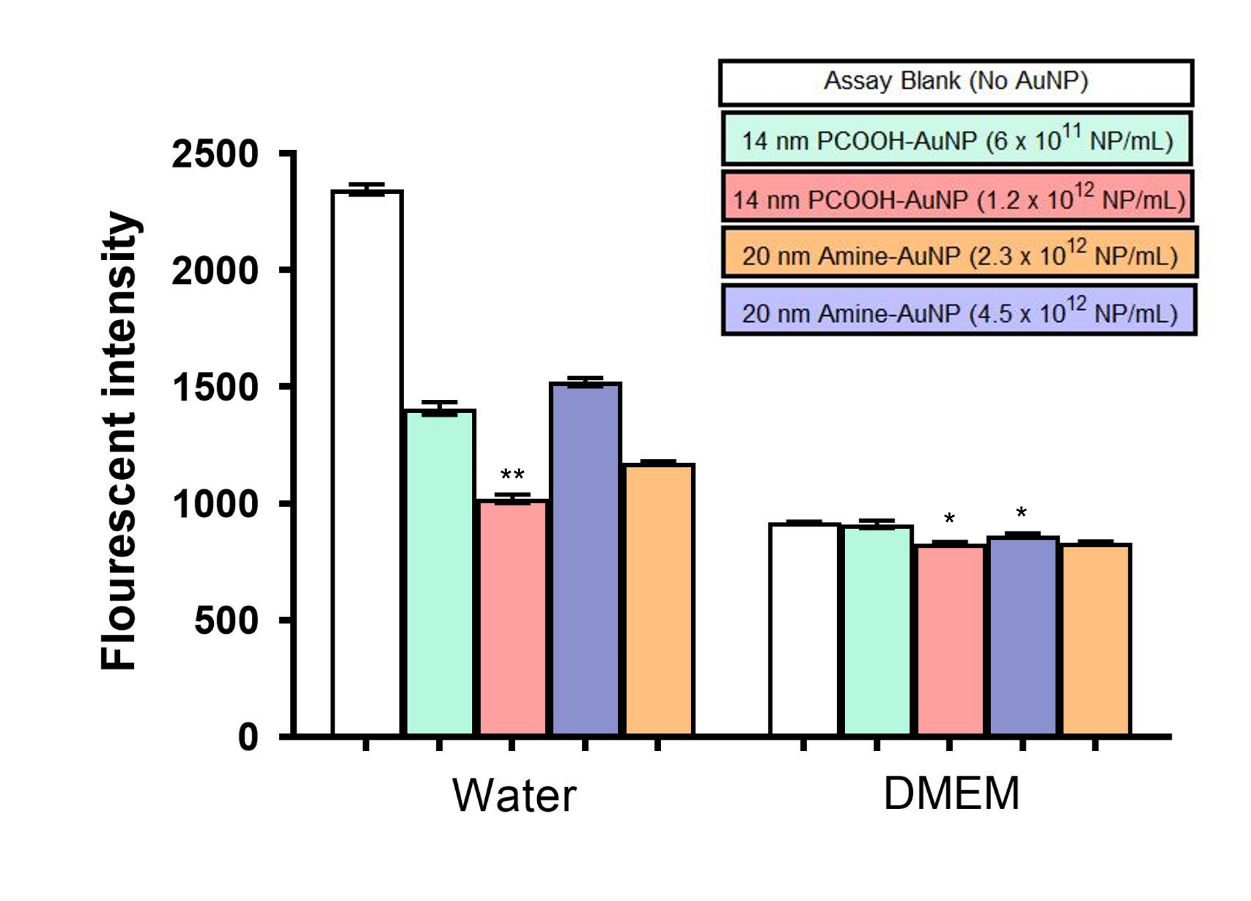


Figure 6: Fluorescent intensity of the caspase-3/7 assay substrate in the presence of gold nanoparticles (AuNP) in water and medium free from foetal calf serum. Blank values were included for reference. The 340±11, 450±50 filter set (nm; excitation, emission wavelengths ± bandpass filter ranges) was used. Significance indicated as *p ≤ 0.05; **p ≤ 0.01.

Discussion and conclusion:

The possibility of NPs interfering with biological assays is one of the major concerns when screening new NPs. Causes of interference can vary greatly and some examples include interference with light absorption, chemical reactions between the NPs and assay substrates, as well as dye adsorption to the NP surface.^1^ Due to the high surface energy of NPs, it has been found that they are able to adsorb dyes to their surface, thereby distorting readings.^2^ It has been found that the stability of some NPs are lower in culture medium than in water.^1^ The DMEM used in this study contained the dye phenol red, which could be a possible explanation for some of the more significant changes in absorbance and fluorescence seen when the AuNPs were diluted in this medium. Culture medium contains various macromolecules that have also been observed to bind to NPs and create protein coronas around the NP, which affects the outcomes of biological assays.^1^

Serum may lower the interaction between cells and NPs as well as the NP uptake into cells, which can affect biological assays results.^3-4^ The DMEM used in the interference studies was not supplemented with FCS, Fobian *et al.* reported low penetration of PCOOH-AuNPs into the MCSs (4.5 µm), indicating the possibility of serum interference with AuNP uptake.^5^ Although some significant interaction was observed for the APH assay, BCA assay, caspase-3/7 activation assay as well as some PI autofluorescence, these were measured with the assumption of full presence of AuNPs, which have been shown to be unlikely by Fobian *et al*.^5^ Furthermore, washing of samples prior to assessment reduces the risk of interference by removal of AuNPs that have not been taken up by the spheroids. Although these will reduce the risk of interference, results will benefit from comparative studies being assessed by label-free means, thus negating the effects of substrate or spectrometric influences.^6^

**References:**

1. Costa C, Brandão F, Bessa MJ, Costa S, Valdiglesias V, Kiliç G, et al. In Vitro Cytotoxicity Of Superparamagnetic Iron Oxide Nanoparticles On Neuronal And Glial Cells. Evaluation Of Nanoparticle Interference With Viability Tests. Journal of Applied Toxicology. 2016; 36(3):361-72.
2. Kroll A, Pillukat MH, Hahn D, Schnekenburger Jr. Interference Of Engineered Nanoparticles With In Vitro Toxicity Assays. Archives of Toxicology. 2012; 86(7):1123-36.
3. Geys J, Nemery B, Hoet PHM. Assay Conditions Can Influence The Outcome Of Cytotoxicity Tests Of Nanomaterials: Better Assay Characterization Is Needed To Compare Studies. Toxicology in Vitro. 2010; 24(2):620-9.
4. Eleonore FH. Cellular Targets And Mechanisms In The Cytotoxic Action Of Non-Biodegradable Engineered Nanoparticles. Current Drug Metabolism, 2013. p. 976-88.
5. Fobian S-F, Petzer M, Vetten M, Steenkamp V, Gulumian M, Cordier W. Mechanisms Facilitating The Uptake Of Carboxyl–Polythene Glycol-Functionalized Gold Nanoparticles Into Multicellular Spheroids. Journal of Pharmacy and Pharmacology. 2022:rgac017.
6. Sanabria NM, Vetten M, Andraos C, Boodhia K, Gulumian M, Antopolsky ME. Gold Nanoparticle Interference Study During The Isolation, Quantification, Purity And Integrity Analysis Of RNA. PLoS ONE2014.
